# Ring-Opening Polymerization of *rac*-β-Butyrolactone Promoted by New Tetradentate Thioether-Amide Ligand-Type Zinc Complexes

**DOI:** 10.3390/polym15224366

**Published:** 2023-11-09

**Authors:** Salvatore Impemba, Gabriele Manca, Irene Tozio, Stefano Milione

**Affiliations:** 1 Center for Colloid and Surface Science, Department of Chemistry, University of Florence, Via della Lastruccia, 3, 50019 Sesto Fiorentino, Firenze, Italy; salimpemba@gmail.com; 2 CNR-ICCOM, Consiglio Nazionale delle Ricerche, Via Madonna del Piano, 10, 50019 Sesto Fiorentino, Firenze, Italy; gmanca@iccom.cnr.it; 3 Department of Chemistry, University of Salerno, Via Giovanni Paolo II, 84084 Fisciano, Salerno, Italy; just_irene@live.it

**Keywords:** polyhydroxyalkanoates, zinc catalyst, ring-opening polymerization

## Abstract

In this work, thioether-amide ligands featuring a combination of hard amide groups with soft donor groups have been employed to develop new zinc catalysts for the ring-opening polymerization of cyclic esters. All complexes were prepared in high yields through alkane elimination reactions with diethyl zinc and characterized using nuclear magnetic resonance (NMR) spectroscopy. Density functional theory (DFT) characterization provided insight into the parameters that influence catalytic activity, such as steric hindrance at the metal center, Lewis acidity and electronic density of thioether-amide ligands. In the presence of one equivalent of isopropanol, all complexes were active in the ring-opening polymerization of *rac*-β-butyrolactone. Quantitative conversion of 100 monomer equivalents was achieved within 1 h at 80 °C in a toluene solution. Number-average molecular weights increased linearly with monomer conversion; the values were in optimal agreement with those expected, and polydispersity index values were narrow and relatively constant throughout the course of polymerization. The most active complex was also effective in the ring-opening polymerization of ε-caprolactone and L-lactide. To propose a reliable reaction path, DFT calculations were undertaken. In the first step of the reaction, the acidic proton of the alcohol is transferred to the basic nitrogen atom of the amide ligand coordinated to the zinc ion. This leads to the alcoholysis of the Zn-N bond and the formation of an alcoholate derivative that starts the polymerization. In subsequent steps, the reaction follows the classical coordination–insertion mechanism.

## 1. Introduction

Polyhydroxyalkanoates (PHAs), a class of naturally occurring polyesters, are attracting remarkable research attention due to their sustainability and potential applications [1,2]. One of the most extensively studied PHAs is poly(3-hydroxybutyrate) (PHB). When obtained from bacterial fermentation, it is a perfectly isotactic polymer with semi-crystalline and thermoplastic behavior. Its mechanical and thermal properties are very similar to those of isotactic polypropylene; thus, it could serve as a green alternative to conventional petroleum-derived plastics [3]. In comparison with starch-derived plastics, PHB displays good resistance to UV light and hydrolytic degradation. However, its application is hindered by several reasons including the high cost of fermentative production, limited scalability of this process and processing difficulties stemming from its high melting temperature [4]. One way to overcome these drawbacks and further its applications is the chemical synthesis of PHB. Undoubtedly, the most promising approach is the ring-opening polymerization (ROP) of β-butyrolactone (β-BL) promoted by a coordination metal complex, as it allows for the effective control of the polymer architectures which, in turn, affect the polymer’s physical properties [5]. Catalytic performance strongly depends on the metal used, as well as on the ancillary ligand coordinated to the metal center. Among the different metals, zinc is advantageous as it is non-toxic, colorless, inexpensive and non-redox-active [6]. Due to these characteristics, many zinc catalysts have been reported for the ROP of lactides and other cyclic esters [7,8,9,10,11]. Worth mentioning are the dinuclear macrocyclic zinc complexes examined by Williams and co-workers as they are the most efficient catalysts for lactide polymerization performed in solution at room temperature (Turnover frequencies (TOFs) up to 60,000 h^−1^) [9].

Despite the great interest in zinc-based catalysts, to date, only few zinc catalysts have been developed for the ROP of β-BL [12,13,14,15,16,17,18,19,20]. One of the first relevant reports was by Coates and co-workers. They found that a β-diiminate zinc alkoxide polymerizes β-BL at high rates under mild conditions: with a [β-BL]/[Zn] ratio of 200, this complex led to a 90% conversion in one hour at room temperature [12]. Rieger and co-workers succeeded in further incrementing the activity of this complex by introducing an electron-withdrawing group on the ligand skeleton [16]. More recently, Rieger and co-workers described a series of aromatic aminotroponiminate zinc complexes and showed that these complexes are able to convert 600 equivalents of β-BL in three hours when combined with 10 equivalents of isopropanol [18]. Du and co-workers reported a series of amido-oxazolinate zinc complexes that were able to convert β-BL in cyclic or linear polymers in the absence or presence of an alcohol [19].

As part of our research interests [21,22,23,24], we have developed a series of group 4 and aluminum metal complexes featuring tetradentate thioether-amide ligands (NSSNs) and studied their activity in the ROP of cyclic esters [25,26,27]. Since these ligands present an intriguing combination of hard and soft donor groups, we thought that they could match the “borderline” soft Lewis acid character of zinc ions, influencing their reactivity positively. In this work, we report the synthesis and characterization of three novel zinc complexes supported by these ligands and their application in the ring-opening polymerization of BBL. These complexes are devoid of labile ligands that can start the polymerization process and are active only in combination with isopropanol, which acts as an activating agent. To shed light on the role of the alcohol and propose a polymerization mechanism, DFT calculations were carried out.

## 2. Results and Discussion

### 2.1. Synthesis and Characterization

The ligands utilized in this work comprise two N-alkyl-aniline linked by a di-thio-ethyl bridge and differ in the substituents bonded to the nitrogen atoms, i.e., isopropyl (NSSN-iPr), cyclohexyl (NSSN-Cy) or mesityl groups (NSSN-Mes). They were synthesized according to procedures found in the literature [25].

**Scheme 1:**
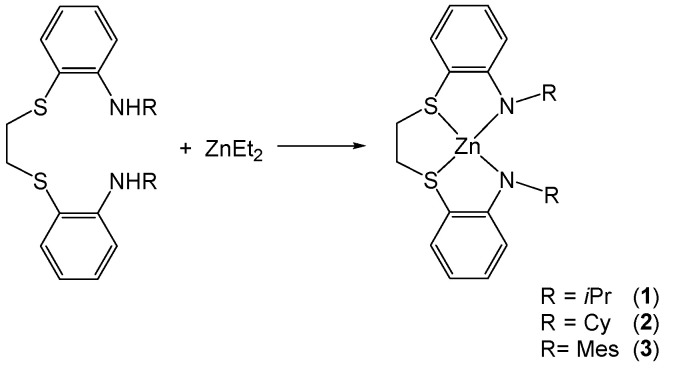
Synthesis of **1**–**3**.

The corresponding zinc complexes **1**–**3** (**1** = (NSSN-iPr)Zn, **2** = (NSSN-Cy)Zn, **3** = (NSSN-Mes)Zn) were prepared by an acid–base reaction between the ligand and one equivalent of diethyl zinc in refluxing benzene, as reported in Scheme 1. The compounds were isolated in high yields (90–98%) as off-white solids. All complexes were characterized using mono- and bidimensional NMR spectroscopy (see Supporting Information).

In the proton spectra, a single set of resonances was observed, indicating that the complexes exist in solution as single isomers. The resonance pattern revealed that all have *C*_2_-symmetric structures: the aniline moieties of the ligands were equivalent, while the protons of the ethyl bridge appeared diastereotopic and gave rise to an AB quartet. No broadening or coalescence of these resonances was observed in the temperature range of 25–80 °C, suggesting that the structures are rigid on the NMR time scale (see Figure S9 in the Supporting Information). Interestingly, the chemical shift difference in the quartet increased as we moved from **1** to **3**, indicating that the rigidity of complexes increases with the bulkiness of the substituents at the nitrogen atoms.

Despite several attempts, we were unable to obtain single crystals suitable for X-ray characterization. To propose reasonable structures, DFT calculations were carried out. In all cases, the binding of the ligands introduced a distortion of the tetrahedral coordination geometry around the zinc center. The values of the computed angles and bond distances are reported in SI. The sulfur and the nitrogen atoms exhibit trigonal pyramidal and trigonal planar geometries, respectively. The *C*_2_-symmetric environment was achieved through the gauche conformation of the SCH_2_CH_2_S bridge.

**Figure 1:**
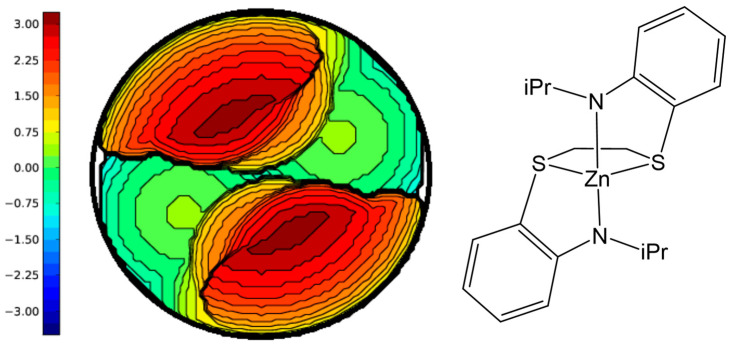
Steric encumbrance map produced by the ligand NSSN-iPr in **1**. The isocontour curves are given in Å.

Lewis acidity and steric hindrance around the zinc metal center are considered two important factors that influence the catalytic activity. The Lewis acidity was assessed by estimating the partial charge of the zinc atom using natural population analysis (NPA). Steric hindrance resulting from the coordinated ligands for **1**–**3** was determined based on the percentage of the total volume occupied by the ligand within a sphere of a 3.5 Å radius centered on the metal core. These calculations were performed using the SambVca 2.1 package, which is freely available and developed by Cavallo et al. [28]. The results are reported in Table 1 (see Supporting Information for more computational result details). The partial charges at the zinc atom are in the range of 1.23–1.39 atomic units (au). These data suggest that the NSSN-iPr and NSSN-Cy ligands transfer a very similar charge density to the metal atom and complex **3** exhibits the lowest acidity in the series. As expected, the ligands occupy a relatively large volume around the metal center, which limits the access to the catalytic pocket (see Figure 1). The %VBur for **1**–**3** increases as the steric encumbrance of the substituents on the nitrogen atoms increases.

### 2.2. Polymerization Studies

Complexes **1**–**3** were assessed as catalysts for the ring-opening polymerization of *rac*-β-butyrolactone under various experimental conditions. All polymerization experiments were carried out in the presence of one equivalent of isopropanol as an activating agent. Complex **1** was inactive in THF or CH_2_Cl_2_, while moderate activity was observed in hexane or chloroform at 60 °C. The highest activity was achieved in a toluene solution at 80 °C; the main results are reported in Table 2. Under these conditions, after 30 min, the monomer conversion reached 84%, resulting in a turnover frequency (TOF) of 167 h^−1^. Complexes **2** and **3** displayed lower activities with TOFs of 155 and 128 h^−1^, respectively. It is worth noting that, under these experimental conditions, complex **1** was inactive in the absence of isopropanol.

**Figure 2:**
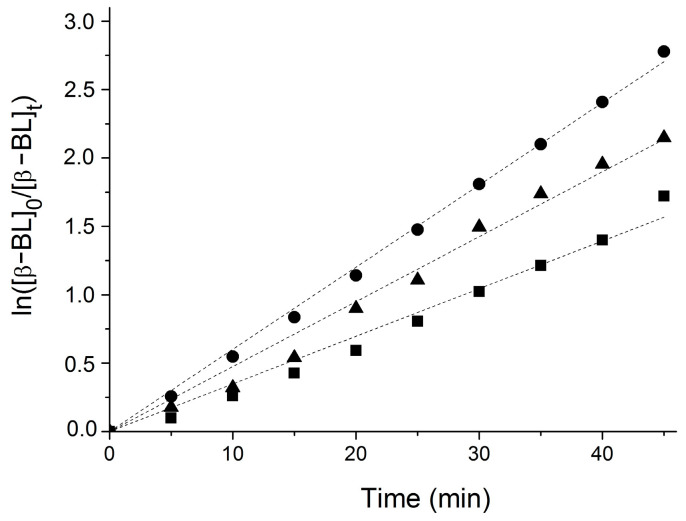
Pseudo-first-order kinetic plots for ROP of β-BL by **1** (*k*_app_ = 0.060 ± 0.001 min^−1^, *R*^2^ = 0.999 ●), **2** (*k*_app_ = 0.047 ± 0.001 min^−1^, *R*^2^ = 0.994, ▲) and **3** (*k*_app_ app = 0.035 ± 0.001 min^−1^, *R*^2^ = 0.992, ■). Conditions: [β-BL]_0_ = 0.69 M; [β-BL]_0_/[cat]_0_ = 100, [iPrOH]_0_/[cat]_0_ = 1, T = 80 °C, toluene (2.0 mL) as solvent.

To gain further insight into the kinetics of the reactions, the polymerizations promoted by **1**–**3**/iPrOH were monitored at regular time intervals, and the corresponding kinetic plots were determined. As shown in Figure 2, in all cases, the polymerization starts instantly, and the reactions proceed with a reaction order of one with respect to the monomer concentration. The apparent propagation rate constants (*k*_app_) were 0.060 ± 0.001 min^−1^, 0.047 ± 0.001 min^−1^ and 0.035 ± 0.001 min^−1^ for **1**, **2** and **3,** respectively. These values follow the same order as observed for the TOFs and confirm that the increase in steric hindrance of the substituent bound to the nitrogen atom has a detrimental effect on the catalytic activities.

**Figure 3:**
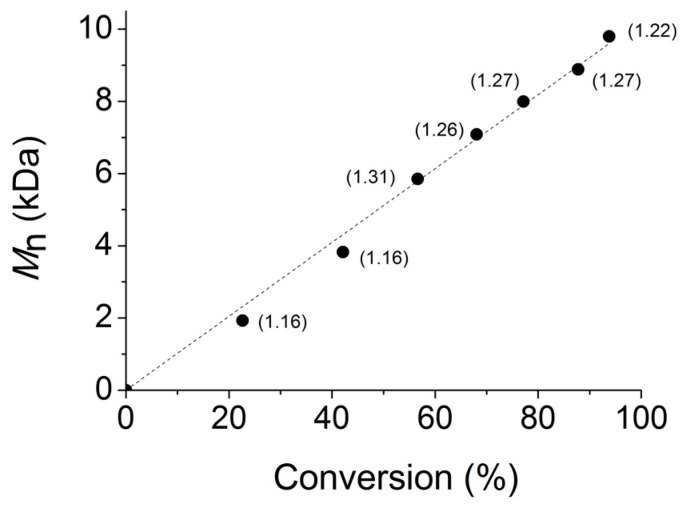
Plot of number-averaged molecular weight *M*_n(exp)_ vs. monomer conversion with theoretical *M*_n(th)_ (dashed line) using **1**/iPrOH as initiator. Conditions: [βBL]_0_ = 0.69 M; [βBL]_0_/[1]_0_ = 100, [iPrOH]_0_/[1]_0_ = 1, T = 80 °C, toluene (2.0 mL) as solvent.

The isolated polymers were subjected to gel permeation chromatography (GPC) to determine the number-average molecular weight (*M*_n(exp)_) and the polydispersity index (*PDI*). The *M*_n(exp)_ values for the polymers obtained by **1**–**3**/iPrOH closely matched those calculated assuming the growth of one polymer chain for one added alcohol equivalent. The *PDI* values exhibited a monomodal and narrow distribution with values in the range of 1.16–1.31. In the case of the polymerization promoted by **1**/iPrOH, the relationship between the *M*_n(exp)_ and monomer conversion was investigated. As reported in Figure 3, the *M*_n(exp)_ increased linearly with conversion, and the values were in excellent agreement with those expected. The *PDI* values were narrow and relatively constant during the polymerization, suggesting that the propagation step was minimally affected by intra- or intermolecular transesterification side reactions. All the data indicate that the polymerization by **1**–**3**/iPrOH proceeds as a controlled ROP process.

Complex **1** was also tested in the ring-opening polymerization of L-lactide (LA) and -caprolactone (εCL), with the main results reported in Table 3. Under the same experimental conditions, the polymerization of LA and εCL proceeded with higher rates consistent with the fact that β-BL, despite its high internal strain, is significantly less reactive. Almost quantitative conversions of LA and εCL were obtained in 30 min and 5 min, respectively. The corresponding TOFs were 182 and 1200 h^−1^. For a better comparison between β-BL and LA, the conversion of LA was monitored during the reaction, and the corresponding kinetic plot was determined (see supporting information). Also in this case, the plots of ln([LA]_0_/[LA]_t_) versus time were linear with the intercept at the origin, indicating that the reaction is first-order in monomer concentration, and no induction period is required before the start of the polymerization. The apparent propagation rate constant was 0.076 ± 0.002 min^−1^, a higher value than that found for the polymerization of β-BL.

Increasing the monomer-to-initiator ratio required a longer reaction time to achieve high monomer conversion. In the case of the εCL polymerization, the catalyst system required fifteen minutes to quantitatively convert 600 monomer equivalents.

Regarding the control of polymerization, the experimental molecular weights of the isolated polymers were in good agreement with those calculated; the polydispersity index values were close to 1. Even for these monomers, the ROP catalytic activity is well-controlled, allowing the production of well-defined polymers.

To gain insights into the reaction mechanism, low-molecular-weight samples of PHB and PLA were prepared by converting 20 monomer equivalents using **1**. In the case of the oligomers of β-BL, the analysis of the ^1^H and ^13^C NMR spectra clearly revealed the presence of signals attributable to isopropoxy and hydroxybutyrate-end groups. Specifically, in the aliphatic region of the ^13^C NMR spectrum, in addition to the four intense peaks due to the main chain repeating butyrate units, two less intense series of peaks were observed (at 69.2/23.1 ppm and at 65.01/43.91/23.0) and assigned to the carbons of the isopropoxyl group and hydroxybutyrate chain end groups, respectively. Minimal or no crotonate end groups were observed in the oligomers. The structure of these oligomers was also supported by MALDI-TOF analysis; the series of peaks were attributable to linear oligomers with the following composition: iPrO[C(=O)CH_2_CH(CH_3_)O]_n_H.

Regarding the terminal groups of the LA oligomers, the ^1^H NMR spectrum showed two quadruplets at 5.04 and 4.33 ppm, assigned to the methine hydrogens of the isopropoxy and to lactyl end groups, respectively. The analysis of the MALDI-TOF spectrum further supported the presence of these end groups. The signals were interspersed by 72 uma, indicating that the propagation step is affected by inter- and intra-transesterification side reactions.

The end group analysis confirms that the reaction starts with the nucleophilic attack of the isopropoxy group on the carbonyl carbon of the monomer and proceeds through the classical coordination–insertion mechanism.

### 2.3. Mechanism of β-BL Polymerization by Zinc Complexes

**Figure 4:**
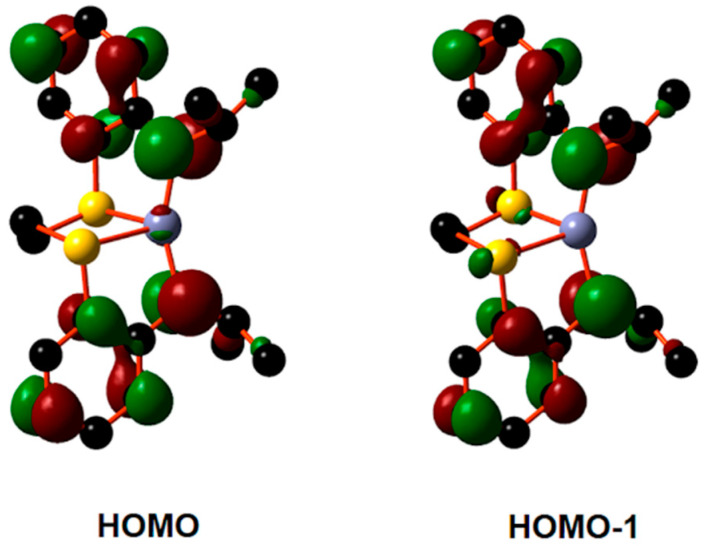
Plot of HOMO and HOMO-1 molecular orbitals of **1**. Hydrogen atoms were hidden for clarity.

A detailed analysis of the electronic structure of **1** revealed that the highest occupied molecular orbital (HOMO) and the HOMO-1 are mainly localized on the lone pairs at the nitrogen centers, as shown in Figure 4.

**Figure 5:**
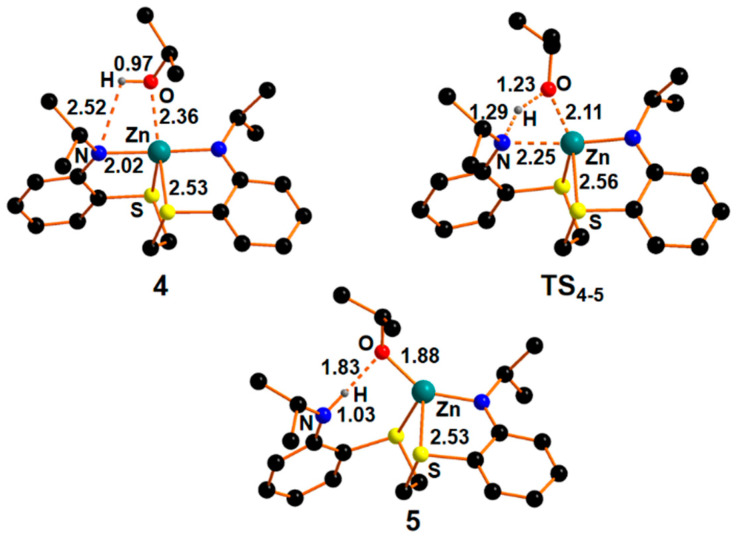
Optimized structures of adduct **4**, transition state **TS_4–5_** and intermediate **5**. Hydrogen atoms, except the involved one, were hidden for clarity.

The presence of electronic density at the amido ligands provides some useful hints to predict that the most plausible first step of the reactivity of complex **1** should be the interaction with the isopropanol species. This aligns with the experimental observation of isopropyl groups as a terminal moiety in the produced polymers. Figure 5 shows the adduct, denoted as **4**, formed between **1** and alcohol with the O-H linkage parallel to the Zn-N bonding. The alcoholic proton seems to be involved in a somewhat weak interaction with the nitrogen center, although the N-H distance is as large as 2.52 Å. The formation of adduct **4** is estimated to occur with a free energy cost of +7.4 kcal mol^−1^. After **4**, the system proceeds through the transition state **TS_4_**_–**5**_, as shown in Figure 5, with a free energy cost of +4.4 kcal mol^−1^. In **TS_4_**_–**5**_, the alcoholic proton lies between the oxygen and the nitrogen centers (O-H and N-H distances are 1.23 and 1.29 Å, respectively) with a NHO angle of 152°. The activation of the O-H bonding leads to the cleavage of the original Zn-N linkage, elongated by 0.23 Å compared to its value in complex **1**. The transition state nature of **TS_4_**_–**5**_ is confirmed by the detection at −1054 cm^−1^ of a single imaginary frequency for the N-H and O-H shortening and lengthening, respectively. The greater the steric encumbrance at the nitrogen ligand, the more hindered the activation of the O-H linkage of isopropanol in the adduct **4**, as occurs for the compound with cyclohexyl and mesythyl substituents.

**Figure 6:**
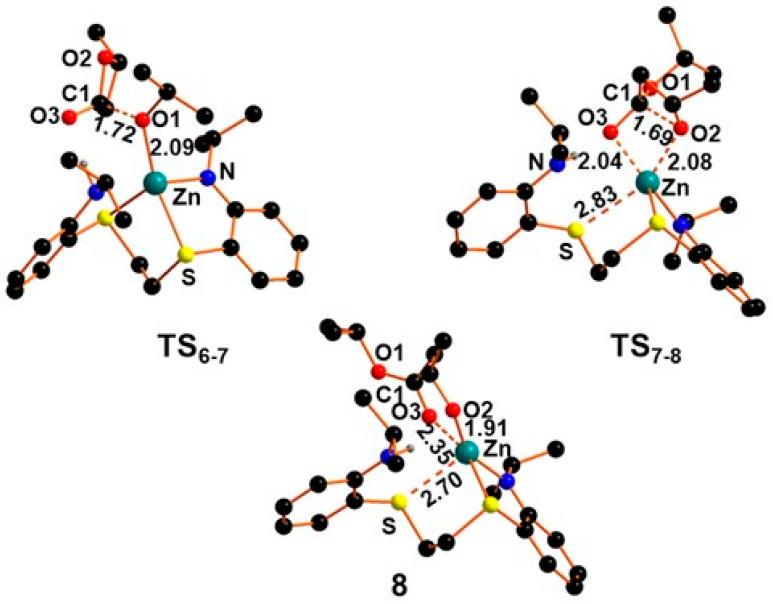
Optimized structure of adduct **TS_6–7_**, transition state **TS_7–8_** and intermediate **8**. Hydrogen atoms, except the involved one, were hidden for clarity.

Intermediate **5** in Figure 5 features a formed Zn-O and N-H linkage with the proton engaged in H-bonding with the oxygen. The free energy gain from **TS_4–5_** has been estimated to be −11.0 kcal mol^−1^. Once formed, the oxygen O1 of the alkoxide ligand can act as a nucleophile toward the carbon center, specifically C1, of the β-butyrolactone, activating it for the polymerization process. An adduct, **6** shown in SI, between **5** and β-butyrolactone has been isolated with a free energy cost of +3.8 kcal mol^−1^; after that, the process evolves through the transition state **TS_6–7_** (shown in Figure 6) with a free energy barrier of +21.4 kcal mol^−1^. The transition state features a O1-C1 of 1.72 Å and an imaginary frequency at −173 cm^−1^ associated with the C1-O1 shortening. Then, intermediate **7**, shown in SI with an already-formed C1-O1 linkage (C1-O1 1.3 Å), is formed with a free energy gain of −13.5 kcal mol^−1^.

**Figure 7:**
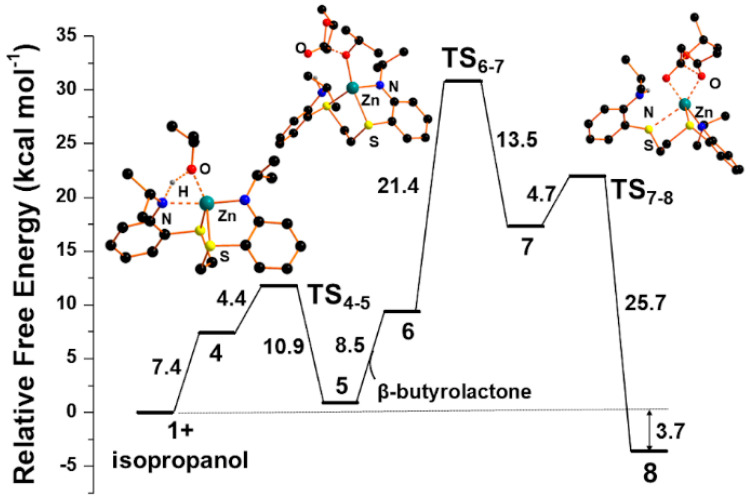
Relative free energy (kcal mol^−1^) pathway for the formation of **8** starting from **1** in the presence of isopropanol and β-butyrolactone.

Intermediate **7** still retains the intact four-membered ring of the β-butyrolactone. Therefore, to promote the polymerization process, a change in coordination to the metal is required. A relaxed scan for the approach of O2 to Zn pointed out the presence of an energy barrier. Consequently, the transition state **TS_7–8_**, shown in Figure 6, has been optimized with a free energy barrier of +4.7 kcal mol^−1^ and a single imaginary frequency at −102 cm^−1^. **TS_7–8_** is characterized by the lengthening of one Zn-S linkage with a length difference between the two Zn-S bonds of ca. 0.24 Å. The O2 coordination to the metal allows the cleavage of the four-membered ring and the formation of intermediate **8** (see Figure 6) with the formation of an open chain and the O2 center able to perform a further β-butyrolactone activation. The formation of **8** starting from transition state TS**_7–8_** has been estimated to be as large as −25.7 kcal mol^−1^. The formation of **8** from **1** and isopropanol and β-butyrolactone has been estimated to be exergonic by −3.7 kcal mol^−1^, as shown in the free energy pathway of Figure 7. The highest free energy barrier of ca. 30.0 kcal mol^−1^ is encountered for the obtainment of intermediate **7** and is in line with the requirement of an experimental temperature not lower than 80 °C. Similar reaction pathways, denoted with a subscript “L”, have been obtained for the activation of L-lactide with comparable free energy barriers, as shown in SI. However, the reactivity of **5** with lactide to provide a similar compound **8** appears somewhat hindered being slightly endergonic by 1.0 kcal mol^−1^.

## 3. Conclusions

In this work, three novel zinc complexes featuring thioether-amide ligands were prepared and characterized using NMR spectroscopy. The k^4^-coordination of the ligands to the metal ion afforded complexes with a distorted tetrahedral geometry whose structures were quite rigid on the NMR time scale.

In the presence of one equivalent of isopropanol, all complexes were able to promote the ring-opening polymerization of *rac*-β-butyrolactone with good activity. The polymerization proceeded in a controlled manner, resulting in polymers with predetermined molecular weights and narrow polydispersities. The most active complex, featuring the least sterically hindered ligand, was also tested in the ring-opening polymerization of ε-caprolactone and L-lactide.

End group analysis indicated that the polymerization follows a coordination–insertion mechanism. To propose a reasonable reaction pathway and elucidate the role of the alcohol, DFT investigations were carried out.

The results achieved in this study demonstrate that thioether-amide ligands are a good platform for the development of effective catalysts for the polymerization of low-reactive cyclic esters, such as β-butyrolactone. The versatility of the ligands permits easy modifications of catalysts’ structures that may improve their activity and lead to ROP catalysts with higher performance.

## 4. Experimental Section

*Materials and methods.* All preparations and subsequent manipulations of air- and/or water-sensitive compounds were carried out under a dry nitrogen atmosphere using a Braun Labmaster drybox or standard Schlenk line techniques. Glassware and vials used in the polymerization were dried in an oven at 120 °C overnight and exposed three times to vacuum-nitrogen cycles. All solvents and reagents used were dried and purified before use. Toluene (Sigma-Aldrich, 99.5%), hexane (Sigma-Aldrich, 99%) were preliminarily dried over CaCl_2_, while THF (Sigma-Aldrich, 99%) was preliminarily treated with potassium hydroxide. Then, all solvents were purified by distillation from sodium under a nitrogen atmosphere. Ligands used for the synthesis of complexes were anhydrificated in vacuum with P_2_O_5_. *Rac*-β-butyrolactone and ε-caprolactone were dried over CaH_2_ one night and freshly distilled under reduced pressure. Lactide was purified by crystallization from dry toluene and then stored over P_2_O_5_. All other chemicals (Sigma-Aldrich) were commercially available and used as received unless otherwise stated. The ligands NSSN-iPr, NSSN-Cy and NSSN-Mes were prepared in accordance with the literature [20].

*Instruments and Measurements.* The NMR spectra were recorded on Bruker Avance 400.13 or Bruker Avance 600 spectrometer at 25 °C, unless otherwise stated. Deuterated solvents were purchased from Cambridge Isotope Laboratories, Inc., degassed and dried over activated 4Å molecular sieves prior to use. Chemical shifts (δ) are listed as parts per million and coupling constants (*J*) in hertz. ^1^H NMR spectra are referenced using the residual solvent peak at δ 7.16 for C_6_D_6_, δ 7.27 for CDCl_3_ and δ 5.32 for CD_2_Cl_2_. ^13^C NMR spectra are referenced using the residual solvent peak at δ 128.39 for C_6_D_6_, δ 77.23 for CDCl_3_ and δ 53.84 for CD_2_Cl_2_. The molecular weights (*M*_n_ and *M*_w_) and the molecular mass distribution (*M*_n_/*M*_w_) of polymer samples were measured by gel permeation chromatography (GPC) at 30 °C, using THF as solvent, flow rate of eluent 1 mL/min, and narrow polystyrene standards as reference. The measurements were performed on a Waters 1525 binary system equipped with a Waters 2414 RI detector using four Styragel columns (range 1000–1,000,000 Å). Every value was the average of two independent measurements.

### 4.1. Synthesis of (NSSN-iPr)Zn (**1**)

A solution of [NSSN-iPr] (0.52 g, 1.44 mmol) in benzene (2.5 mL) was added to a stirred benzene solution of Zn(C_2_H_5_)_2_ (0.18 g, 1.44 mmol in 2.5 mL of benzene). The resulting yellow solution was refluxed for 24 h, after which the volatile byproducts and the solvent were removed under vacuum. The crude product was washed with hexane (10 mL) to give **1** as a yellow solid (0.55 g, 90%). ^1^H NMR (400.13 MHz, C_2_D_2_Cl_4_, 25 °C): δ 6.27–7.31 (m, 8H, ArH), 3.73 (m, 2H, CH), 3.10 (m, 2H, S-CH_2_), 2.77 (m, 2H, S-CH_2_), 1.31 (m, 12H, 4 × CH_3_). ^13^C NMR (100.62 MHz, C_2_D_2_Cl_4_, 25 °C): δ 24.48, 25.67, 38.98, 46.85, 109.14, 111.07, 111.66, 132.09, 136.16, 156.66.

### 4.2. Synthesis of (NSSN-Cy)Zn (**2**)

Complex **2** was prepared from [NSSN-Cy] (0.47 g, 1.07 mmol) and Zn(C_2_H_5_)_2_ (0.13 g, 1.07 mmol), as described above for **1**. Yield: 0.53 g (98%). ^1^H NMR (400.13 MHz, C_2_D_2_Cl_4_, 25 °C): δ 6.26–7.32 (m, 8H, ArH), 3.29 (m, 2H, CH), 3.10 (m, 2H, S-CH_2_), 2.21 (m, 2H, S-CH_2_), 1.28–2.07 (m, 20H, CH_3_+CH_2_ cyclohexyl group). ^13^C NMR (100.62 MHz, C_2_D_2_Cl_4_, 25 °C): δ 25.65, 25.87, 26.13, 35.36, 36.42, 39.05, 55.58, 109.12, 110.91, 111.55, 132.02, 136.25, 156.38.

### 4.3. Synthesis of (NSSN-Mes)Zn (**3**)

Complex **3** was prepared from [NSSN-Mes] (1.03 g, 2.01 mmol) and Zn(C_2_H_5_)2 (0.25 g, 2.01 mmol), as described above for **1**. Yield: 1.10 g (95%). ^1^H NMR (400.13 MHz, CD_2_Cl_2_, 25 °C): δ 5.88–7.41 (m, 12H, ArH), 3.86 (m, 2H, S-CH_2_), 2.40 (m, 2H, S-CH_2_), 2.25 (s, 6H, 2 × CH_3_), 2.11 (s, 6H, 2 × CH_3_), 1.42 (s, 6H, 2 × CH_3_). ^13^C NMR (100.62 MHz, CD_2_Cl_2_, 25 °C): δ 17.48, 18.83, 20.97, 41.37, 110.83, 112.81, 112.88, 129.23, 129.50, 132.59, 133.58, 134.91, 135.69, 136.57, 144.04, 156.04.

### 4.4. Typical Procedure for Cyclic Ester Polymerization

In a glovebox, a Schlenk flask (10 cm^3^) was charged sequentially with the monomer (*rac-*β-butyrolactone or L-lactide), catalyst and solvent (2 mL). The mixture was heated thermostatically at the required temperature. At specified time intervals, a small amount of the polymerization mixture was sampled by using a pipette and quenched in wet CDCl_3_. This fraction was subjected to monomer conversion determination, which was monitored by the integration of monomer versus polymer methine resonances in the ^1^H NMR spectrum (CDCl_3_) at 25 °C. After the required polymerization time, the reaction mixture was quenched with wet n-hexane. The obtained polymer was collected by filtration and dried in a vacuum at 40 °C for 16 h.

### 4.5. Computational Details

All the obtained structures were isolated using the B97D [29] functional within the Gaussian 16 package [30] and were validated as minima or transition states by the vibrational frequency calculations. The experimental solvent, toluene, was taken into account within the CPCM model [31]. The Stuttgart–Dresden pseudo-potential was used for the zinc center, while the Triple Zeta def2tzvp basis set was adopted for all the other atomic species. The atomic charges were calculated within ATP theory [32]. The coordinates of all the optimized structures as well as their main energetic features are available on request. The buried volume calculations were performed with the SambVca 2.1 package, a piece of software free of charge developed by Cavallo et al. [28]. The radius of the sphere around the metal center was set to 3.5 Å, while we adopted the Bondi radii scaled by 1.17 for the atoms, and a mesh of 0.1 Å was used to scan the sphere for buried voxels.

## Figures and Tables

**Table 1:** Summary of DFT results.

Complex	Zn Charge *^(a)^*	%VBur *^(b)^*	Zn-N *^(c)^*	S-Zn-N *^(d)^*
**1**	1.39	74.3	1.94	86.1
**2**	1.39	78.4	1.93	86.0
**3**	1.23	83.2	1.93	85.2

*^(a)^* Charge of the zinc atom determined through NPA (au). *^(b)^* Percent buried volume. *^(c)^* Bond length (Å). *^(d)^* Angle of bond (°).

**Table 2:** Ring-opening polymerization of *rac*-β-butyrolactone promoted by **1**–**3**/iPrOH.

Entry *^(a)^*	Catalyst	Time (min)	Conv. *^(b)^* (%)	TOF *^(c)^* (h^−1^)	*M*_n(th)_ *^(d)^*	*M*_n(expt)_ *^(e)^*	*PDI ^(e)^*
1	**1**	30	84	168	7.2	7.2	1.17
2	**2**	30	77	154	6.6	6.0	1.13
3	**3**	30	64	128	5.5	5.2	1.21
4	**1**	60	97	97	8.3	8.2	1.22
5	**2**	60	90	90	7.7	7.5	1.23
6	**3**	60	90	90	7.7	7.3	1.91

*^(a)^* All reactions were carried out in 2.0 mL of toluene as solvent, [cat]_0_ = 6.9 mM, [β-BL] = 0.69 M, [β-BL]_0_/[iPrOH]_0_/[cat]_0_ = 100/1/1. *^(b)^* Molecular conversion determined by ^1^H NMR spectroscopy (CDCl_3_, 298 K). *^(c)^* TOF = mol_B-BL_/(mol_Zn_ h). *^(d)^* Calculated molecular weight using *M*_n(th)_ (kg mol^–1^) = (86.09 × ([β-BL]_0_/[iPrOH]_0_) × conversion)/1000. *^(e)^* Experimental molecular weight *M*_n(expt)_ (kg mol^–1^) and polydispersity index (*PDI*) determined by GPC in THF using polystyrene standards and corrected using the factor 0.54.

**Table 3:** Ring-opening polymerization of L-lactide (LLA) and ε-caprolactone (εCL) promoted by **1/**iPrOH.

Entry *^(a)^*	Monomer	[mon]/[1]	Time (min)	Conv. *^(c)^* (%)	TOF *^(d)^* (h^−1^)	*M*_n(th)_ *^(e)^*	*M*_n(expt)_ *^(f)^*	*PDI ^(f)^*
	LLA	100	30	90	180	13.0	11.9	1.19
7 *^(b)^*	LLA	250	120	95	406	34.2	30.3	1.25
8	εCL	100	5	99	1200	11.3	9.8	1.15
9	εCL	300	10	99	1930	33.8	31.1	1.28
10	εCL	600	15	99	2600	67.7	62.5	1.32

*^(a)^* All reactions were carried out in 2.0 mL of toluene as solvent, [1]_0_ = 6.9 mM, [1]_0_/[iPrOH]_0_ = 1/1, temperature = 80 °C. *^(b)^* [1]_0_ = 2.8 mM. *^(c)^* Molecular conversion determined by ^1^H NMR spectroscopy (CDCl_3_, 298 K). *^(d)^* TOF = mol_mon_/(mol_Zn_ h). *^(e)^* Calculated molecular weight using *M*_n(th)_ (kg mol^–1^) = (144.13 × ([mon]_0_/[iPrOH]_0_) × conversion)/1000. *^(f)^* Experimental molecular weight *M*_n(expt)_ (kg mol^–1^) and polydispersity index (*PDI*) determined by GPC in THF using polystyrene standards and corrected using the factor 0.58 for PLA and 0.56 for PCL.

## Data Availability

The data that support the findings of this study are available from the corresponding author, upon reasonable request.

## Supplementary Materials

The following supporting information can be downloaded at https://www.mdpi.com/article/10.3390/polym15224366/s1, Figures giving NMR spectra of proligands and complexes. Figures giving NMR spectra of oligomers of PBL and PLA. Relative free energy pathway for the formation of 8L starting from 1 together with isopropanol and LLA. Cartesian coordinates and free energies of all the structures optimized in the computational analysis.
