# Supplementary material for: Ring-Opening Polymerization of rac-β-Butyrolactone Promoted by New Tetradentate Thioether-Amide Ligand-Type Zinc Complexes
Source: Polymers (Basel). 2023 Nov 9;15(22):4366. doi: 10.3390/polym15224366 (PMC10675234; doi:10.3390/polym15224366)
Supplement: Supplementary file 1 [file polymers-15-04366-s001.zip › polymers-2682627-supplementary.pdf]

Supplementary Information

Ring-Opening Polymerization of *rac*-β-Butyrolactone Promoted by New Tetradentate Thioether-Amide Ligand-Type Zinc Complexes

*Salvatore Impemba [1], Gabriele Manca [2], Irene Tozio [3] and Stefano Milione [3,]**

[1] Center for Colloid and Surface Science, Department of Chemistry, University of Florence, Via della Lastruccia, 3, 50019 Sesto Fiorentino, Firenze, Italy

[2] CNR-ICCOM, Consiglio Nazionale delle Ricerche, Via Madonna del Piano, 10, 50019 Sesto Fiorentino, Firenze, Italy

[3] Department of Chemistry, University of Salerno, Via Giovanni Paolo II, 84084 Fisciano, Salerno, Italy

Contents

	pag.
**Experimental section**	*S3*
Figure S1: ^{1}H NMR of [NSSN-iPr].	*S5*
Figure S2: ^{13}C NMR of [NSSN-iPr].	*S5*
Figure S3: ^{1}H NMR of [NSSN-iPr]Zn (**1**).	*S6*
Figure S4: ^{13}C NMR of [NSSN-iPr]Zn (**1**).	*S6*
Figure S5: Dept 135 NMR of [NSSN-iPr]Zn (**1**).	*S7*
Figure S6: HSQC of [NSSN-iPr]Zn (**1**).	*S7*
Figure S7: Variable temperature ^{1}H NMR of [NSSN-iPr]Zn (**1**).	*S8*
Figure S8: ^{1}H NMR of [NSSN-Cy].	*S9*
Figure S9: ^{13}C NMR of [NSSN-Cy].	*S9*
Figure S10: ^{1}H NMR of [NSSN-Cy]Zn (**2**).	*S10*
Figure S11: ^{13}C NMR of [NSSN-Cy]Zn (**2**).	*S10*
Figure S12: Comparison between ^{13}C and Dept 135 NMR of [NSSN-Cy]Zn (**2**).	*S11*
Figure S13: HSQC and COSY of the aromatic zone of [NSSN-Cy]Zn (**2**).	*S11*
Figure S14: ^{1}H NMR of [NSSN-Mes].	*S12*
Figure S15: ^{13}C NMR of [NSSN-Mes].	*S12*
Figure S16: ^{1}H NMR of [NSSN-Mes]Zn (**3**).	*S13*
Figure S17: ^{13}C NMR of [NSSN-Mes]Zn (**3**)	*S14*
Figure S18: Comparison between ^{13}C and Dept 135 NMR of [NSSN-Mes]Zn (**3**)	*S15*
Figure S19: Pseudo-first-order kinetic plots for ROP of LA by **1.**	*S16*
Figure S20: ^{1}H NMR of PBL obtained using [NSSN-iPr]Zn (**1**) as catalyst.	*S17*
Figure S21: ^{13}C NMR of PBL obtained using [NSSN-iPr]Zn (**1**) as catalyst.	*S17*
Figure S22: ^{1}H NMR of oligomers of PBL obtained using [NSSN-iPr]Zn (**1**) as catalyst.	*S18*
Figure S23: ^{13}C NMR of oligomers of PBL obtained using [NSSN-iPr]Zn (**1**) as catalyst.	*S18*
Figure S24: ^{1}H NMR of oligomers of PLA obtained using [NSSN-iPr]Zn (**1**) as catalyst	*S19*
Figure S25: ^{13}C NMR of oligomers of PLA obtained using [NSSN-iPr]Zn (**1**) as catalyst	*S19*
Figure S26: SEC of PBL obtained using [NSSN-iPr]Zn (**1**) as catalyst.	*S20*
Figure S27: Minimum-energy structure of complex **1**.	*S21*
Figure S28: Minimum-energy structure of complex **2**.	*S21*
Figure S29: Minimum-energy structure of complex **3**.	*S22*
Figure S30: Minimum-energy structure of adduct **6**.	*S22*
Figure S31: Minimum-energy structure of intermediate **7**.	*S23*
Figure S32. Relative free energy pathway for the formation of $\mathbf{8_L}$ starting from **1** together with isopropanol and LLA.	*S23*
Cartesian coordinates and free energies of all the structures optimized in the computational analysis.	*S24*

.

Experimental section

Materials and methods. All preparations and subsequent manipulation of air- and/or water-sensitive compounds were carried out under a dry nitrogen atmosphere using a Braun Labmaster drybox or standard Schlenk line techniques. Glassware and vials used in the polymerization were dried in an oven at 120°C overnight and exposed three times to vacuum-nitrogen cycles. All solvents and reagent were dried and purified before use. Toluene (Sigma-Aldrich, 99.5%) and hexane (Sigma-Aldrich, 99%) were heated to reflux for 48 h over sodium or sodium ketyls and distilled before use for moisture- and oxygen-sensitive reactions. All other solvents were used as received (TCI or Sigma-Aldrich) or distilled under reduced pressure over calcium hydride. Ligands used for the synthesis of complexes were dried in vacuum with P_2O_5. Rac-β-butyrolactone (BBL), ε-caprolactone (ε-CL) and isopropyl alcohol (iPrOH) were dried over CaH_2 one night and freshly distilled under reduced pressure. L-lactide was purified by recrystallization from toluene twice and subsequently dried over P_2O_5 under dynamic vacuum and finally stored in the glove box. Deuterated solvents were purchased from Sigma-Aldrich dried over activated 4Å molecular sieves prior to use.

Instruments and Measurements. The NMR spectra were collected by using Bruker Avance spectrometers (600, 400, 300 MHz for ^{1}H). Chemical shifts (δ) are listed as parts per million and coupling constants (*J*) in Hertz. ^{1}H NMR spectra are referenced using the residual solvent peak at δ 7.16 for C_6H_6, δ 7.27 for $CDCl_3$, δ 5.32 for CD_2Cl_2 and δ 6.00 for $C_2D_2Cl_4$. ^{13}C NMR spectra are referenced using the residual solvent peak at δ at 128.39 for C_6H_6, δ 77.23 for $CDCl_3$, δ 53.84 for CD_2Cl_2 and δ 73.78 for $C_2D_2Cl_4$.

Synthesis of the NSSN ligands.

Synthesis of 1,2-Bis(aminophenylthio)ethane [NSSN]. 1,2-Dibromoethane (7.3 g, 39 mmol) was added dropwise to a solution of 2-aminothiophenol (9.8 g, 78 mmol) and sodium hydroxide (3.1 g, 78 mmol) in ethanol (100 mL). The reaction mixture was refluxed for 1 h. Upon completion of the reaction, the reaction mixture was cooled to room temperature and the solvent was removed in vacuo. Water (50 ml) was added, and the reaction mixture was extracted with diethyl ether (4x 50 mL). The organic layer was dried with anhydrous Na_2SO_4, filtered and evaporated to dryness in vacuo to get a yellow solid product (10.7 g, yield = 99 %). ^{1}H NMR (400.13 MHz, $CDCl_3$, 25 °C) δ ppm. 6.64-7.31 (m, 8H, ArH), 4.35 (s, 4H, 2x NH_2), 2.86 (s, 4H, 2x S-CH_2). ^{13}C NMR (100.62 MHz, $CDCl_3$, 25 °C) δ ppm. 34.61, 115.16, 116.86, 118.71, 130.15, 136.30, 148.68.

Synthesis of 2,2'-(ethane-1,2-diylbis(sulfanediyl))bis(N-isopropylaniline) [NSSN-iPr]. [NSSN] (5.79 g, 21 mmol), zinc (13.73 g, 0.21 mol), acetic acid (60 mL), and acetone (13.46 g, 0.21 mol) were added to a 250 mL round-bottom one necked flask equipped with a condenser and a Teflon-sealed stirbar. The mixture was heated to room temperature for 48 h. After it was cooled to room temperature, the mixture was quenched with a 30% NH_3 aqueous solution (150 mL) and diethyl ether (200 mL). The organic layer was dried with anhydrous $MgSO_4$, and a whitish solid product was obtained upon removal of the solvent (6.7 g, 88%). ^{1}H NMR (400.13 MHz, CD_2Cl_2, 25 °C) δ ppm. 6.54-7.33 (m, 8H, ArH), 4.94 (s, 2H, 2x NH), 3.63-3.67 (q, 2H, 2x CH), 2.77 (s, 4H, 2x S-CH_2), 1.23

(d, J = 6.38 Hz, 12H, 4x CH_3). ^{13}C NMR (100.62 MHz, CD_2Cl_2, 25 °C) δ ppm. 22.57, 34.51, 43.91, 110.56, 115.93, 115.97, 130.22, 136.52, 148.65.

Synthesis of 2,2'-(Ethane-1,2-diylbis(sulfanediyl))bis(N-cyclohexylaniline) [NSSN-Cy]. [NSSN] (6.67 g, 24 mmol), zinc (15.8 g, 0.24 mol), acetic acid (100 mL), and cyclohexanone (9.48 g, 48 mmol) were added to a 250 mL round bottom one-necked flask equipped with a condenser and a Teflon sealed stirbar. The mixture was heated to 65 °C for 48 h. After it was cooled to room temperature, the mixture was quenched with a 30% NH_3 aqueous solution (200 mL) and diethyl ether (300 mL). The organic layer was dried with anhydrous Na_2SO_4, and a whitish solid product was obtained upon removal of the solvent (10.3 g, 97%).^{1}H NMR (400.13 MHz, $CDCl_3$, 25 °C) δ ppm. 6.52-7.35 (m, 8H, ArH), 5.01 (s, 2H, 2x NH), 3.27-3.30 (m, 2H, 2x CH), 2.77 (s, 4H, 2x S-CH_2), 1.21-2.02 (m, 20H, cyclohexyl). ^{13}C NMR (100.62 MHz, $CDCl_3$, 25 °C) δ ppm. 25.08, 26.10, 33.35, 34.83, 51.48, 110.75, 110.76, 116.18, 130.50, 136.96, 148.72.

2,2'-(Ethane-1,2-diylbis(sulfanediyl))bis(N-2,4,6- trimethylaniline), [NSSN-Mes]. A Schlenk flask was charged with [NSSN] (0.6 g, 2.2 mmol), mesityl bromide (0.8 g, 4.0 mmol), tris(dibenzylideneacetone)dipalladium(0) (0.078 g, 0.085 mmol), rac-2,2′-bis(diphenylphosphino)-1,1′-binaphthyl (0.13 g, 0.21 mmol), sodium tert-butoxide (0.63 g, 6.56 mmol), and toluene (15 mL). The reaction mixture was stirred and heated to 110 °C under a stream of N_2 for 72 h. After it was cooled to room temperature, the mixture was quenched with a saturated NH_4Cl aqueous solution and extracted with methylene chloride. The organic layer was dried with anhydrous $MgSO_4$ and concentrated to dryness under reduced pressure to afford a brown oil. This product was purified via flash column chromatography (SiO_2, 230−400 mesh, 4:1 n-hexane/CH_2Cl_2 as the eluent) to give a white solid (0.5 g, 45%).^{1}H NMR (400.13 MHz, CD_2Cl_2, 25 °C) δ ppm. 6.03-7.39 (m, 12H, ArH), 6.41 (s, 4H, 2x NH), 3.01 (s, 4H, 2x S-CH_2), 2.29 (s, 6H, *p*-CH_3), 2.06 (s, 12H, *o*-CH_3). ^{13}C NMR (100.62 MHz, CD_2Cl_2, 25 °C) δ ppm. 18.31, 21.06, 34.79, 111.35, 116.23, 117.67, 129.50, 130.61, 135.53, 136.25, 136.62, 136.79, 148.29.

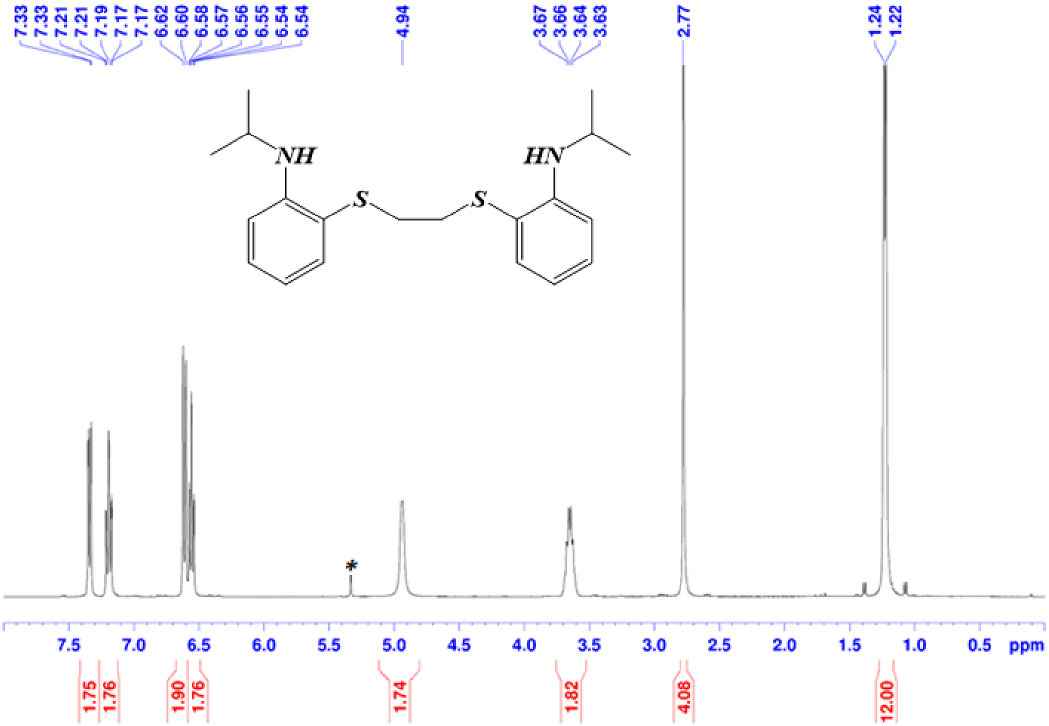

Figure S1: ^{1}H NMR of [NSSN-iPr] (400.13 MHz, $^{*}CD_2Cl_2$, 25 °C).

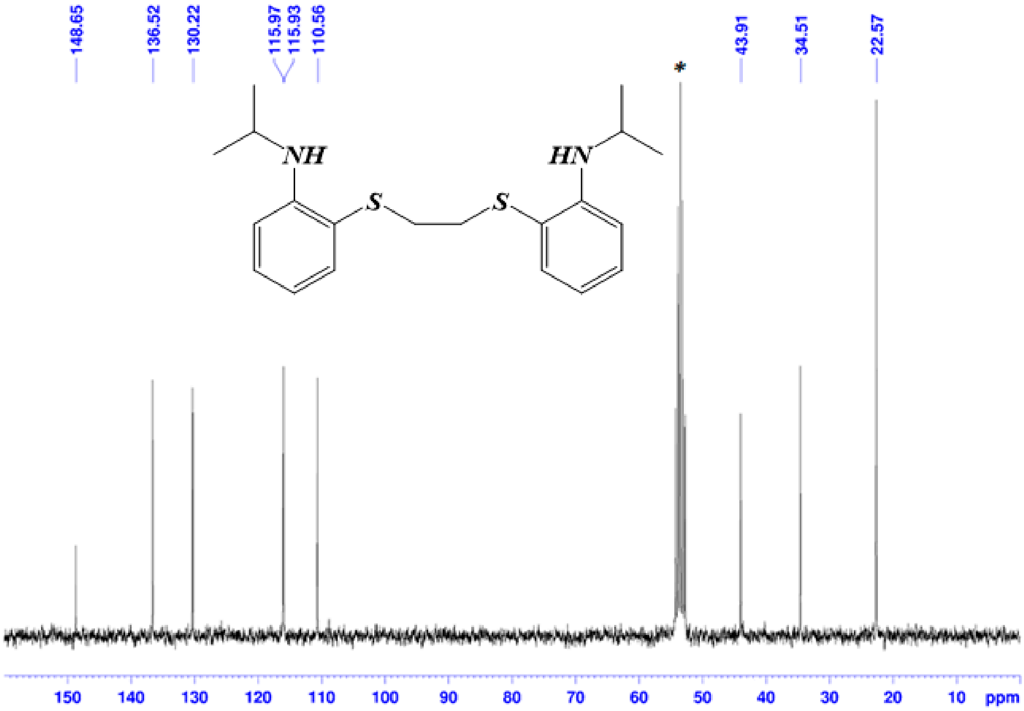

Figure S2: ^{13}C NMR of [NSSN-iPr] (100.62 MHz, $^{*}CD_2Cl_2$, 25 °C).

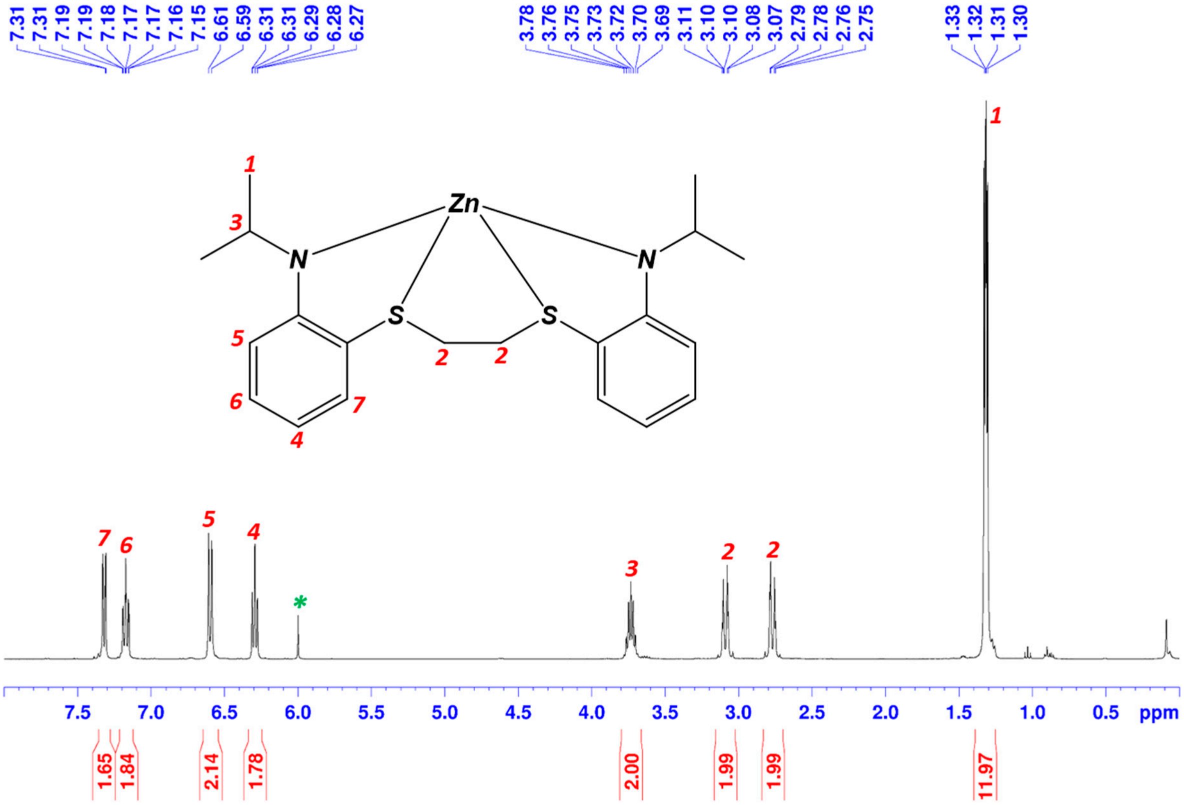

Figure S3: ^{1}H NMR of [NSSN-iPr]Zn (**1**) (400.13 MHz, $^{*}C_2D_2Cl_4$, 25 °C).

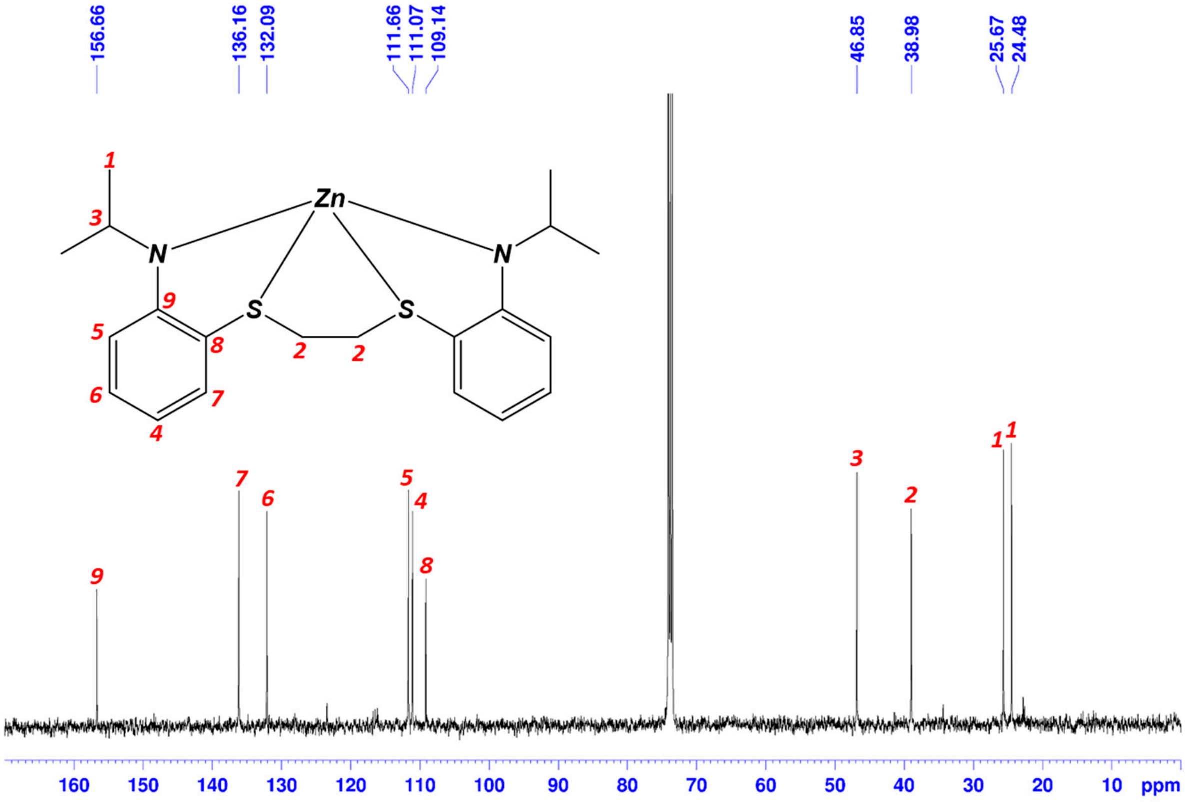

Figure S4: ^{13}C NMR of [NSSN-iPr]Zn (**1**) (100.62 MHz, $^{*}C_2D_2Cl_4$, 25 °C).

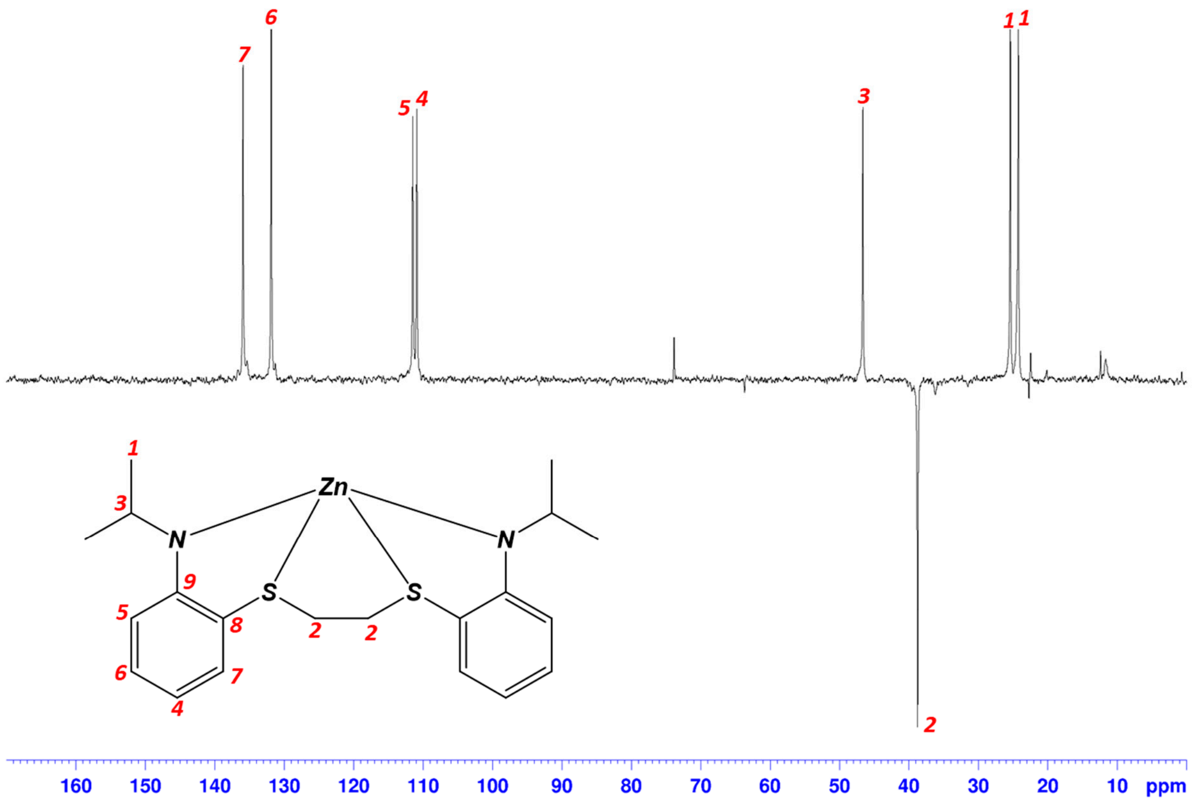

Figure S5: Dept 135 NMR of [NSSN-iPr]Zn (**1**) (100.62 MHz, *$C_2D_2Cl_4$, 25 °C).

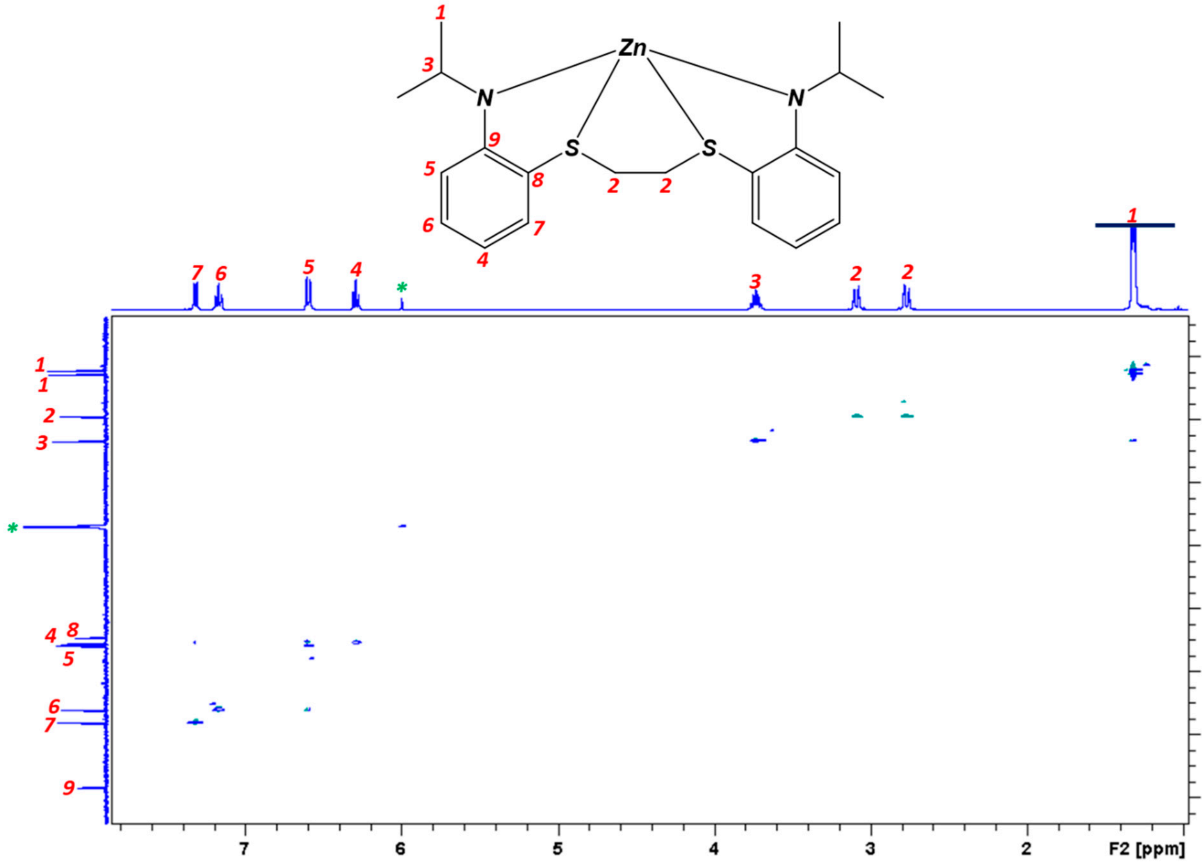

Figure S6: HSQC of [NSSN-iPr]Zn (**1**) (*$C_2D_2Cl_4$, 25 °C).

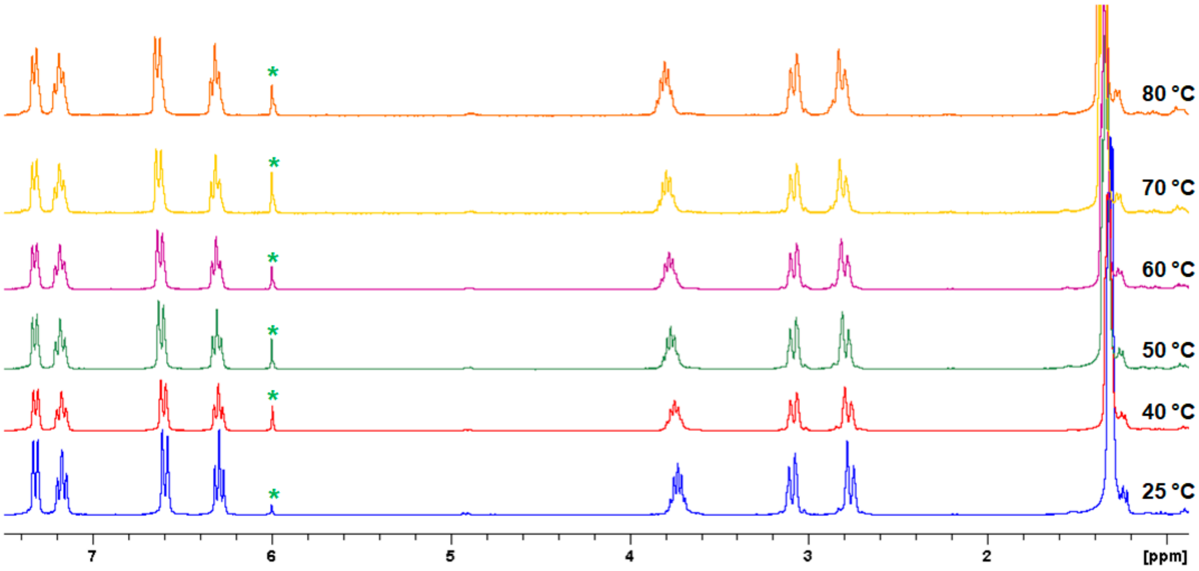

Figure S7: Variable temperature ^{1}H NMR of [NSSN-iPr]Zn (**1**) (*400.13 MHz, *$C_2D_2Cl_4$).

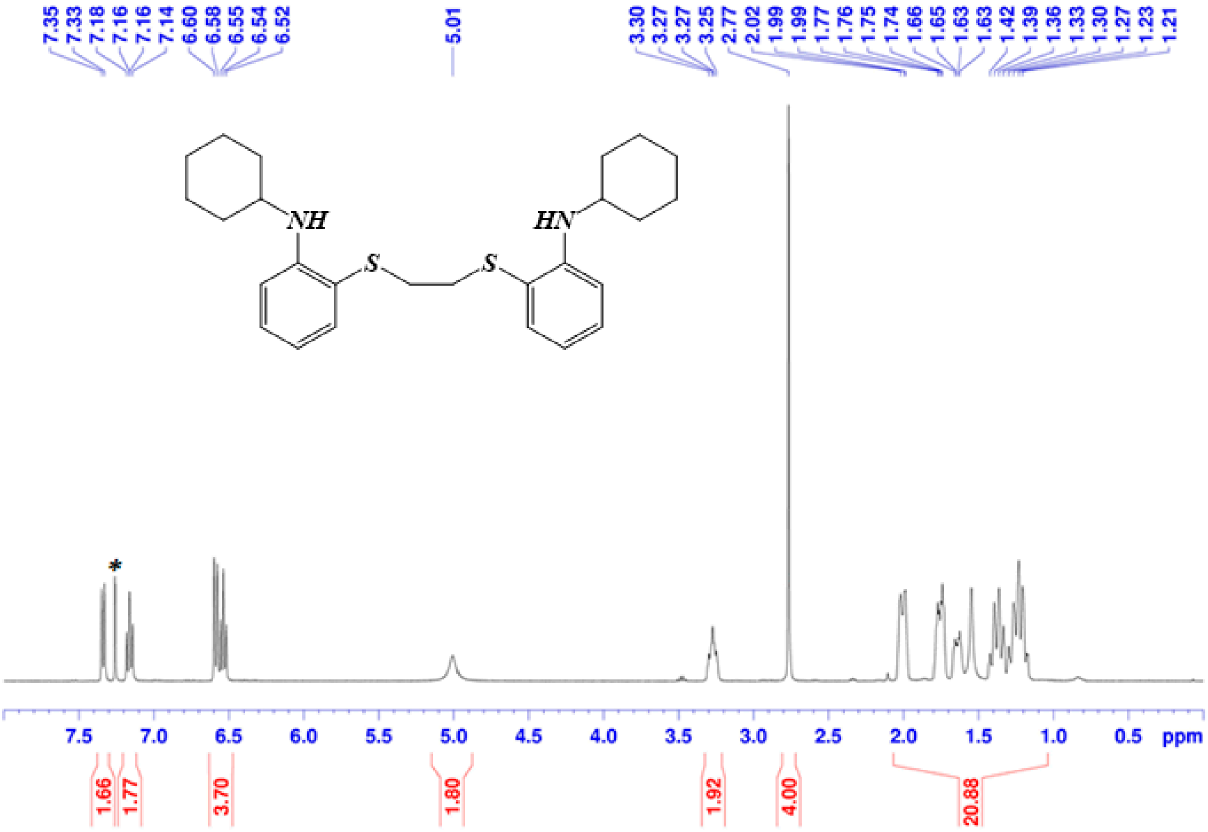

Figure S8: ^{1}H NMR of [NSSN-Cy] (400.13 MHz, *CDCl$_3$, 25 °C).

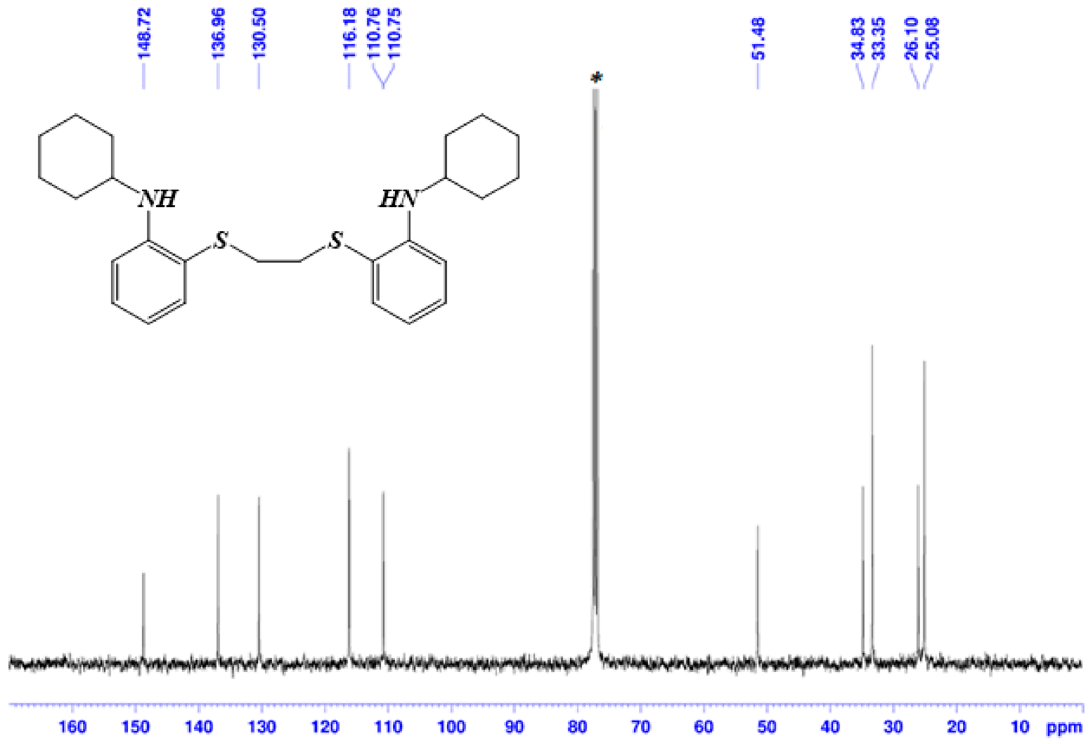

Figure S9: ^{13}C NMR of [NSSN-Cy] (100.62 MHz, *CDCl$_3$, 25 °C).

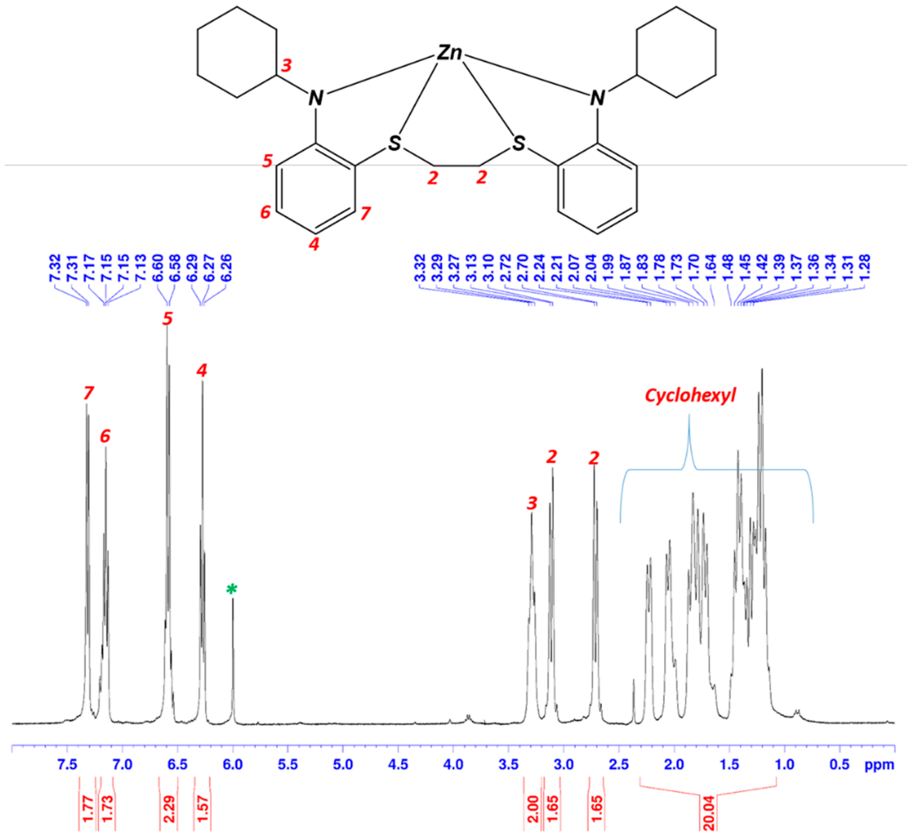

Figure S10: ^{1}H NMR of [NSSN-Cy]Zn (**2**) (400.13 MHz, $^{*}C_2D_2Cl_4$, 25 °C).

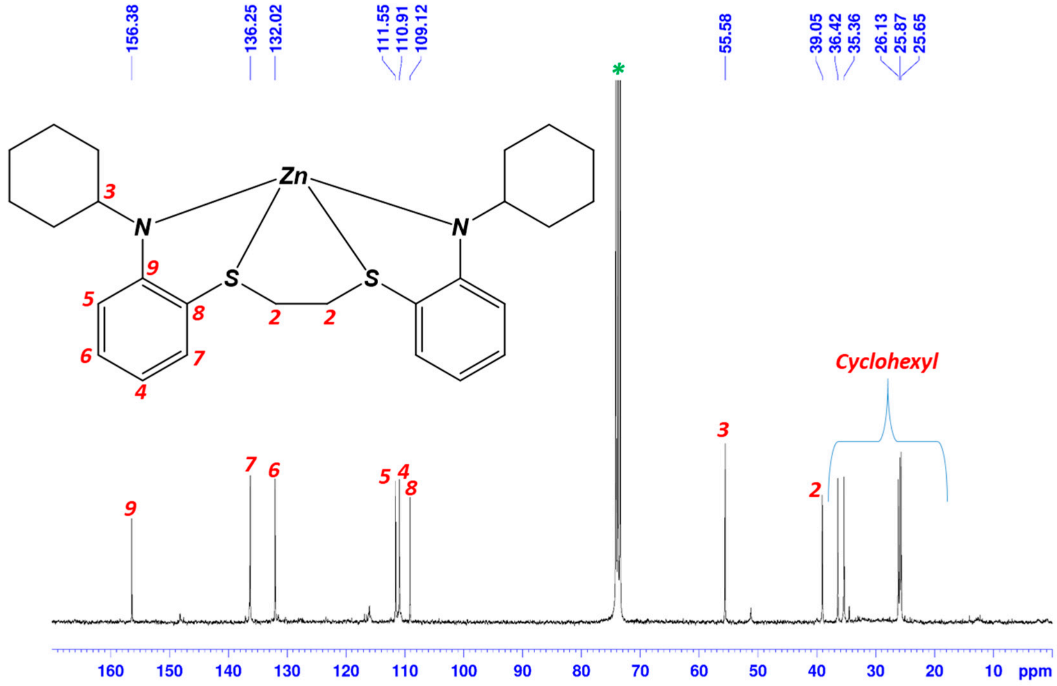

Figure S11: ^{13}C NMR of [NSSN-Cy]Zn (**2**) (100.62 MHz, $^{*}C_2D_2Cl_4$, 25 °C).

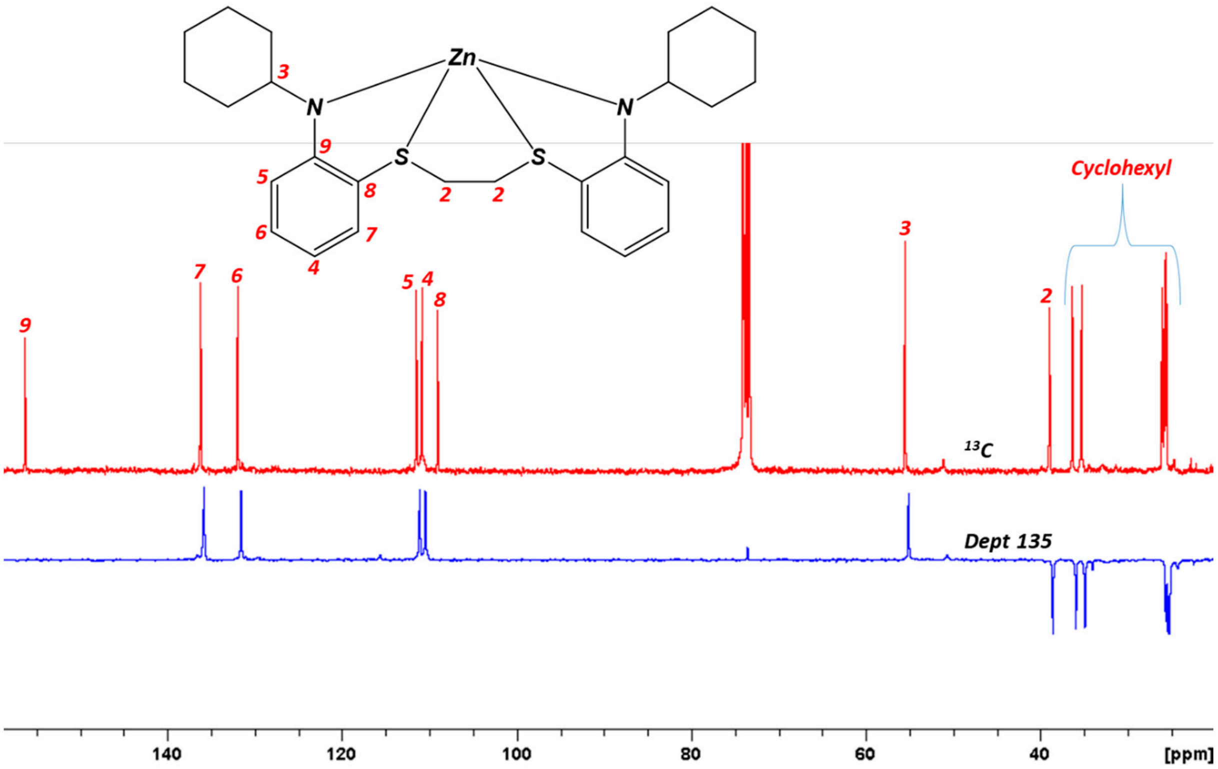

Figure S12: Comparison between ^{13}C and Dept 135 NMR of [NSSN-Cy]Zn (**2**) (100.62 MHz, $^{*}C_2D_2Cl_4$, 25 °C).

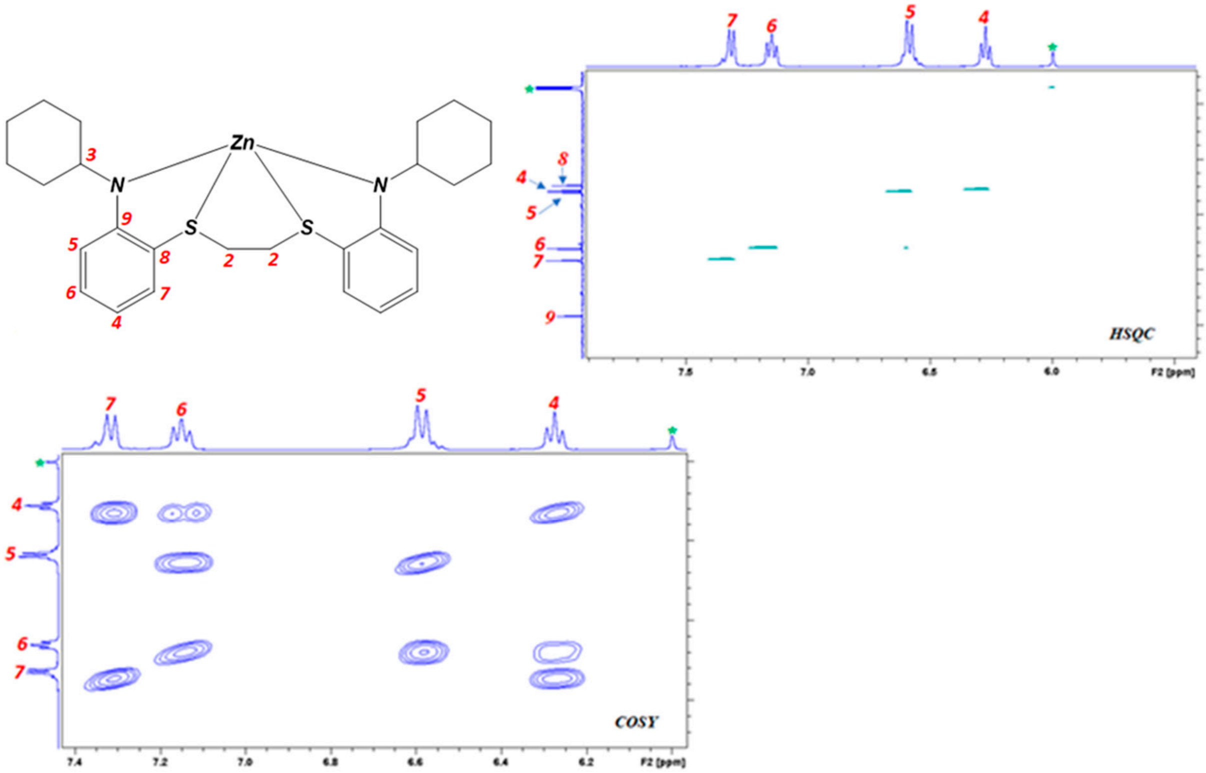

Figure S13: HSQC and COSY of the aromatic zone of [NSSN-Cy]Zn (**2**) ($^{*}C_2D_2Cl_4$, 25 °C).

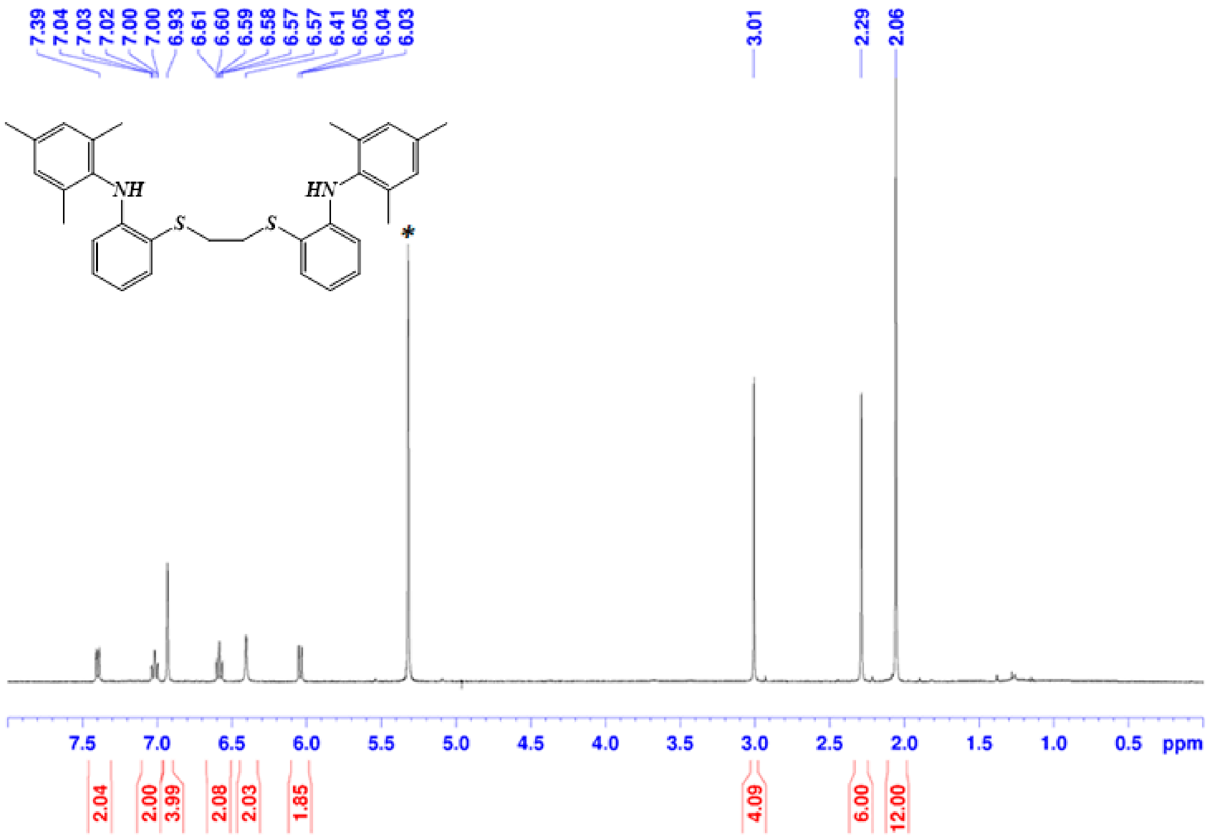

Figure S14: ^{1}H NMR of [NSSN-Mes] (400.13 MHz, $^{*}CD_2Cl_2$, 25 °C).

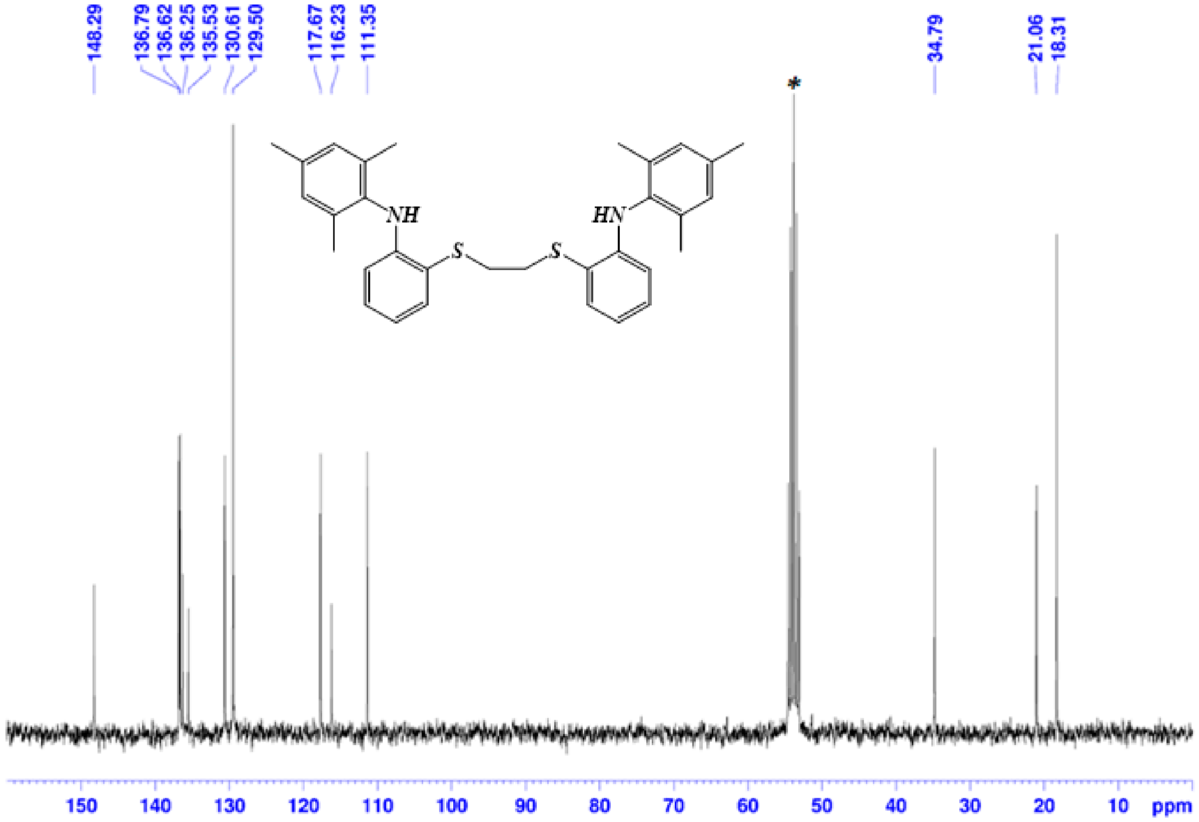

Figure S15: ^{13}C NMR of [NSSN-Mes] (100.62 MHz, $^{*}CD_2Cl_2$, 25 °C).

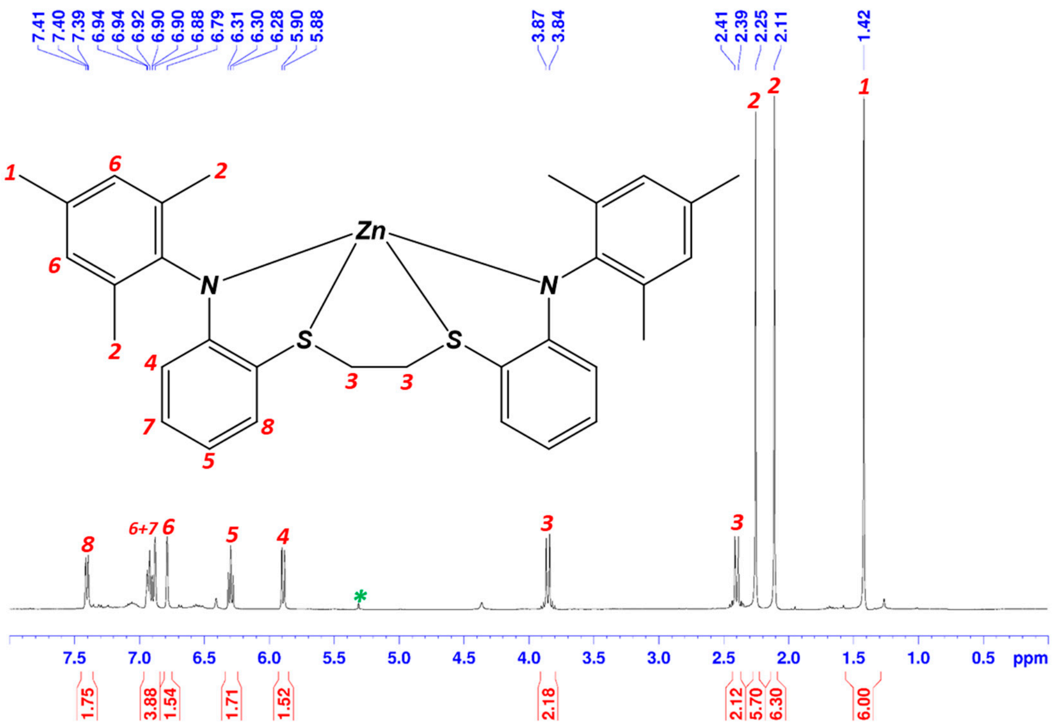

Figure S16: ^{1}H NMR of [NSSN-Mes]Zn (**3**) (400.13 MHz, $^{*}CD_2Cl_2$, 25 °C).

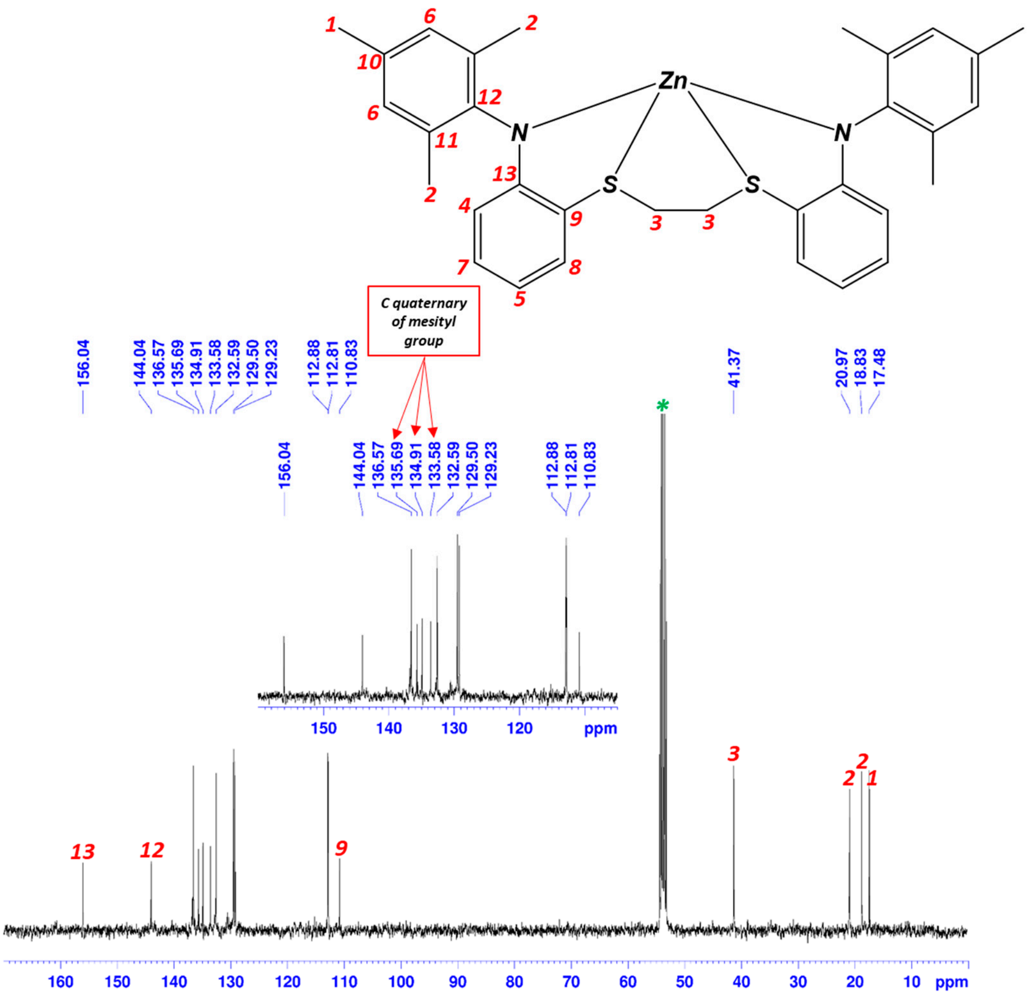

Figure S17: ^{13}C NMR of [NSSN-Mes]Zn (**3**) (100.62 MHz, $^{*}CD_2Cl_2$, 25 °C).

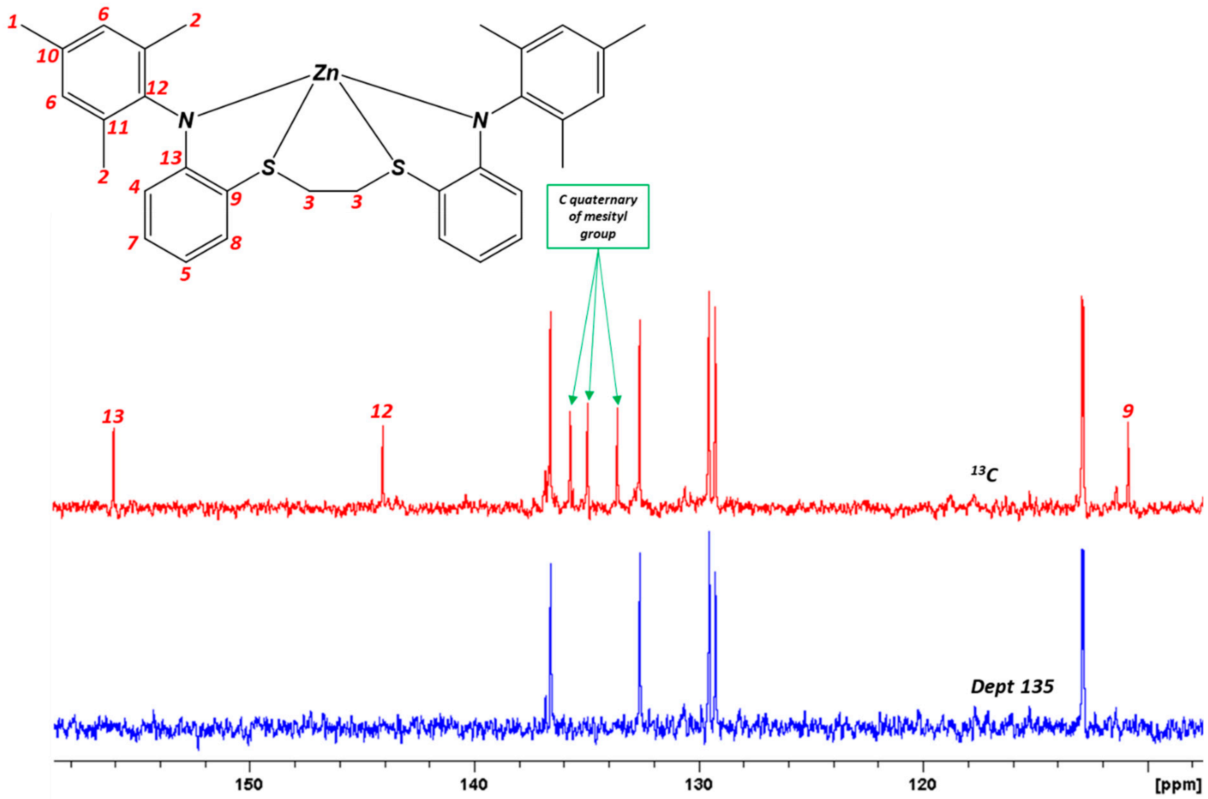

Figure S18: comparison between ^{13}C and Dept 135 NMR of the aromatic zone of [NSSN-Mes]Zn (**3**) (100.62 MHz, $^{*}CD_2Cl_2$, 25 °C).

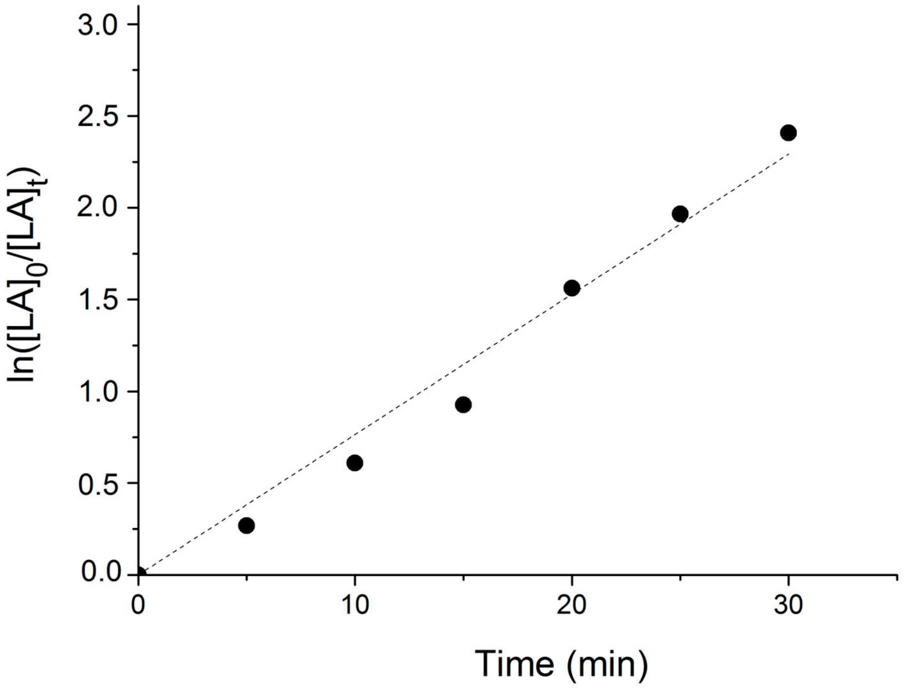

Figure S19: Pseudo-first-order kinetic plots for ROP of LA by **1** (k_{app}= 0.076 ± 0.002 min^{-1}, R^2= 0.991 ●). Conditions: $[LA]_0$= 0.69 M; $[\beta BL]_0/[cat]_0$= 100, $[iPrOH]_0/[cat]_0$= 1, T= 80 °C, toluene (2.0 ml) as solvent.

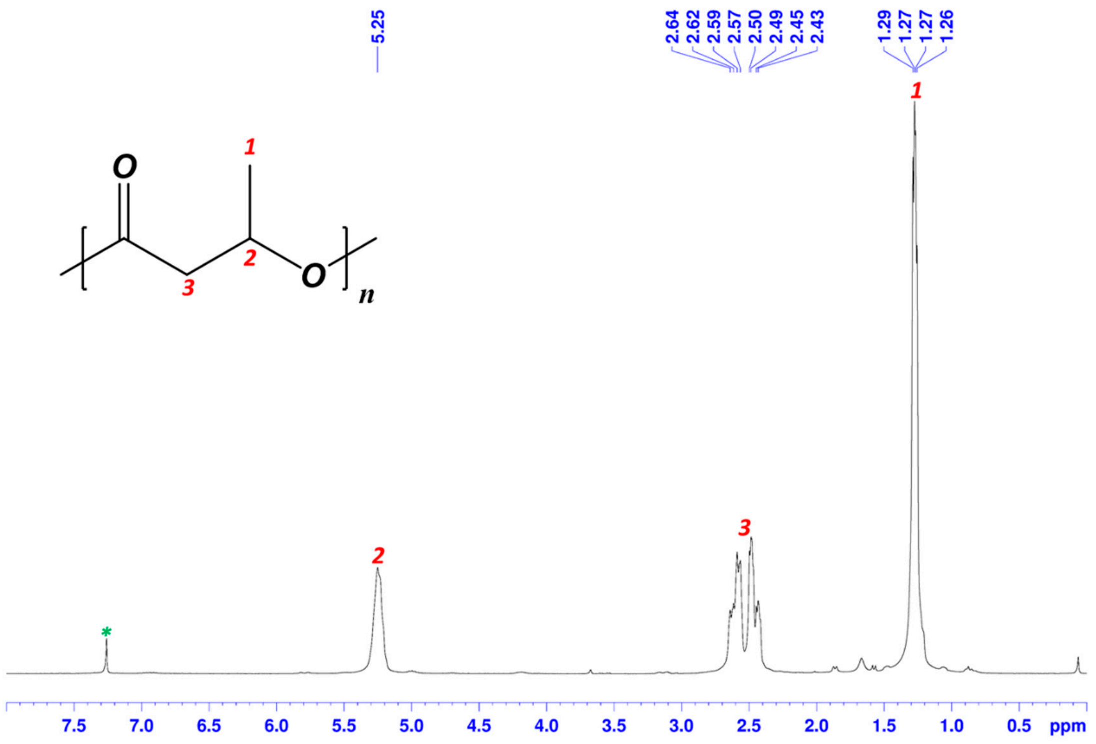

Figure S20: ^{1}H NMR of PBL obtained using [NSSN-iPr]Zn (**1**) as catalyst (Table 2, Entry 1). The small peaks at 5.8 and 6.9 ppm can be assigned to the alkenic protons in trans-crotonate group, which is presumably generated during the precipitation of the polymer product. (400.13 MHz, *$CDCl_3$, 25°C).

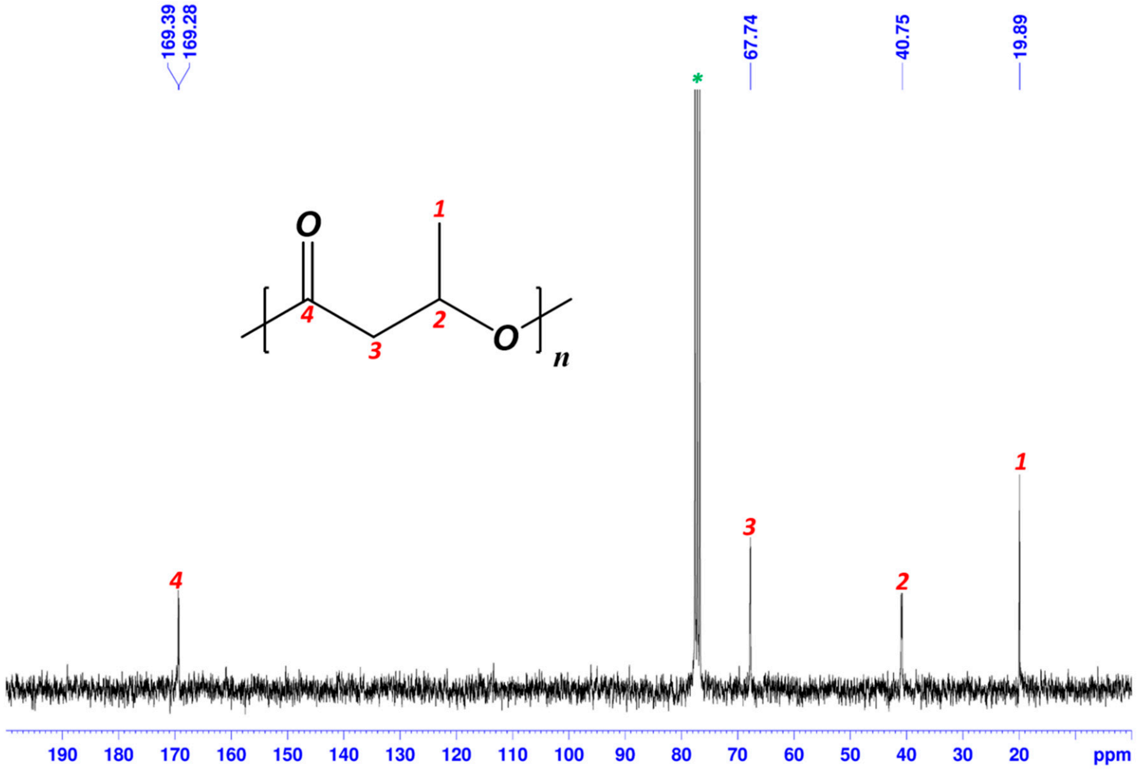

Figure S21: ^{13}C NMR of PBL obtained using [NSSN-iPr]Zn (**1**) as catalyst (Table 2, Entry 1), (100.62 MHz, *$CDCl_3$, 25°C).

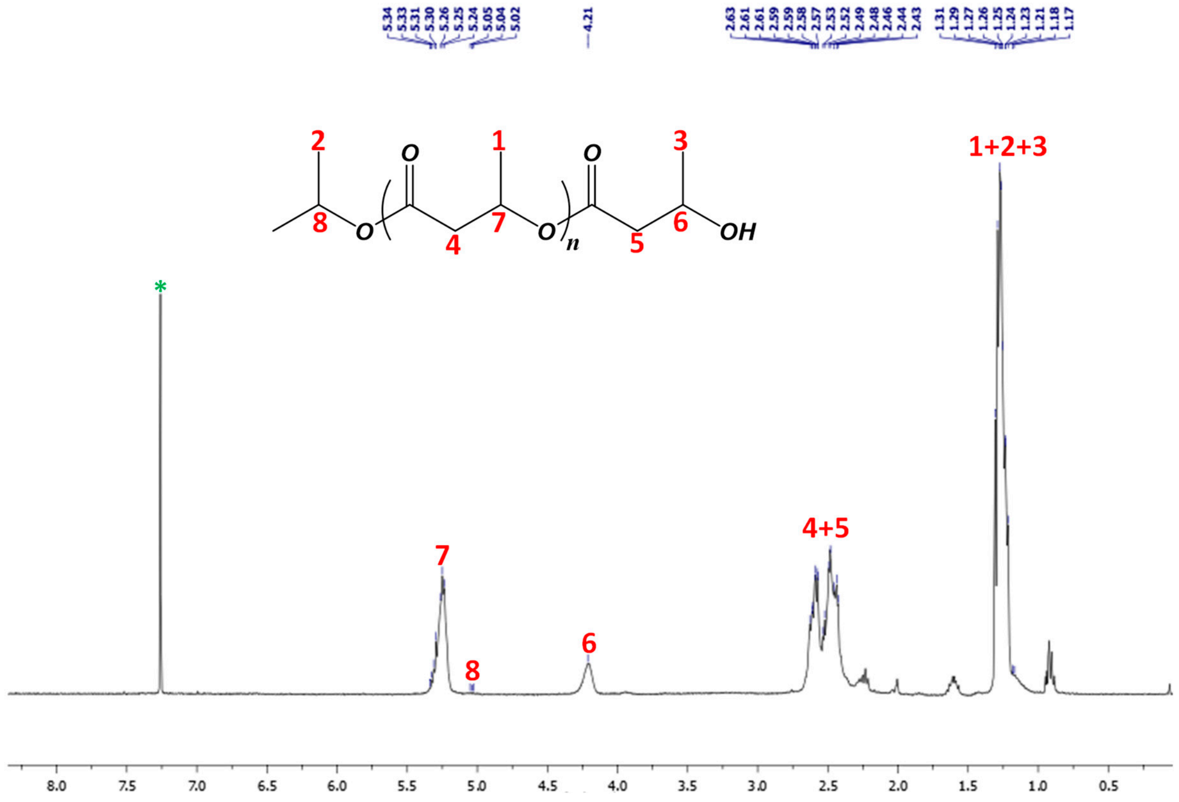

Figure S22: ^{1}H NMR of oligomers of PBL obtained using [NSSN-iPr]Zn (**1**) as catalyst. $M_{n(NMR)}$ = 300 Da, $M_{n(GPC)}$ = 315 Da. (400.13 MHz, *$CDCl_3$, 25°C).

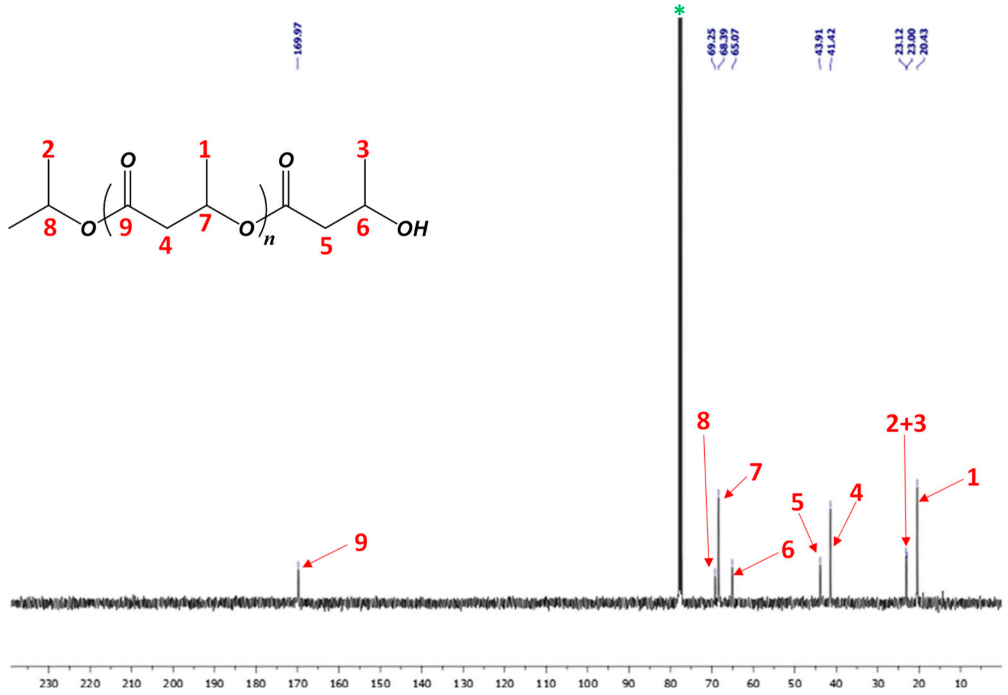

Figure S23: ^{13}C NMR of oligomers of PBL obtained using [NSSN-iPr]Zn (**1**) as catalyst (100.62 MHz, *$CDCl_3$, 25°C).

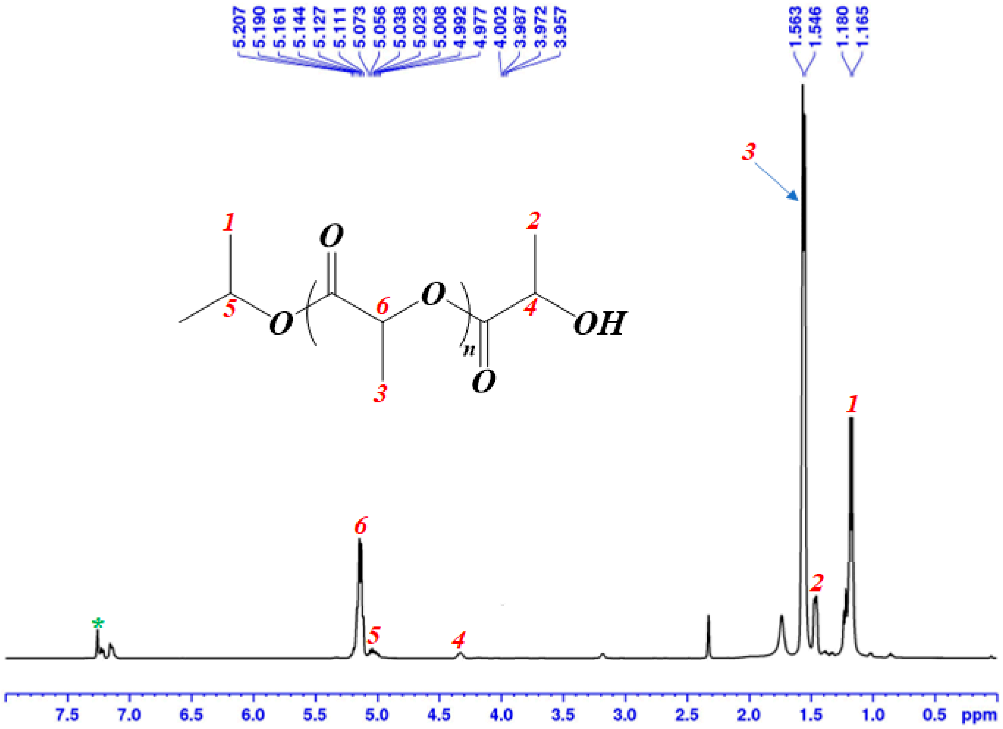

Figure S24: ^{1}H NMR of oligomers of PLA obtained using [NSSN-iPr]Zn (**1**) as catalyst (400.13 MHz, *CDCl3, 25°C).

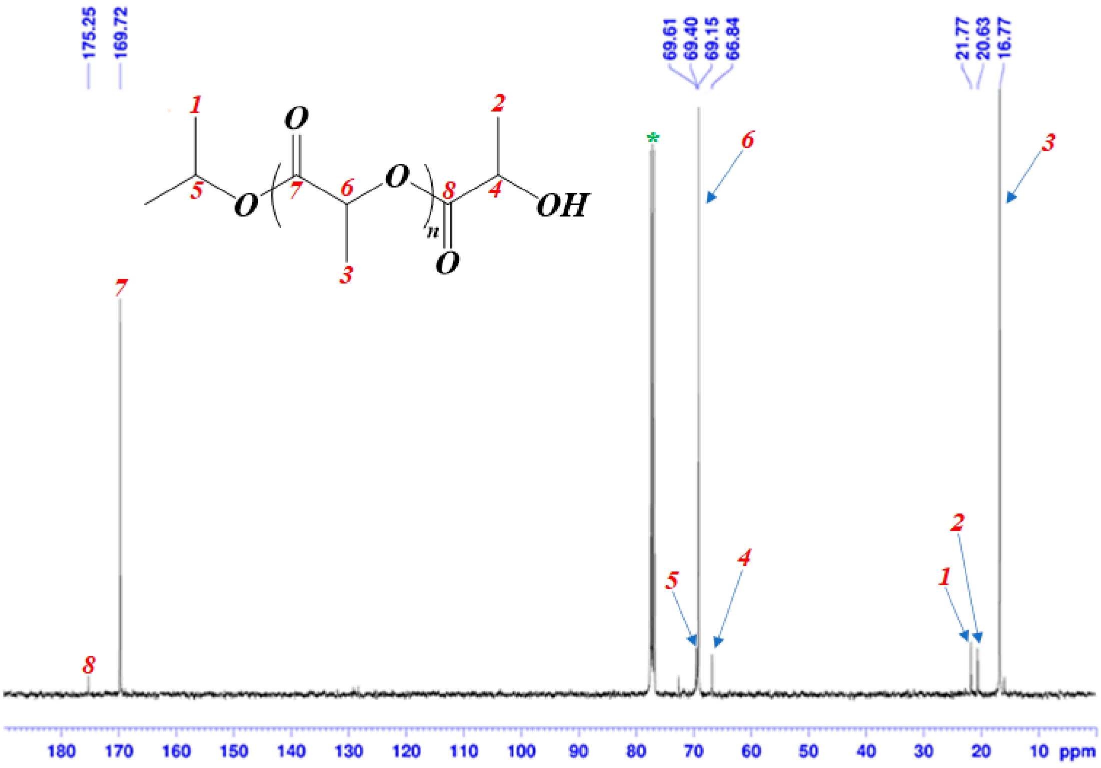

Figure S25: ^{13}C NMR of oligomers of PLA obtained using [NSSN-iPr]Zn (**1**) as catalyst (100.62 MHz, *CDCl3, 25°C).

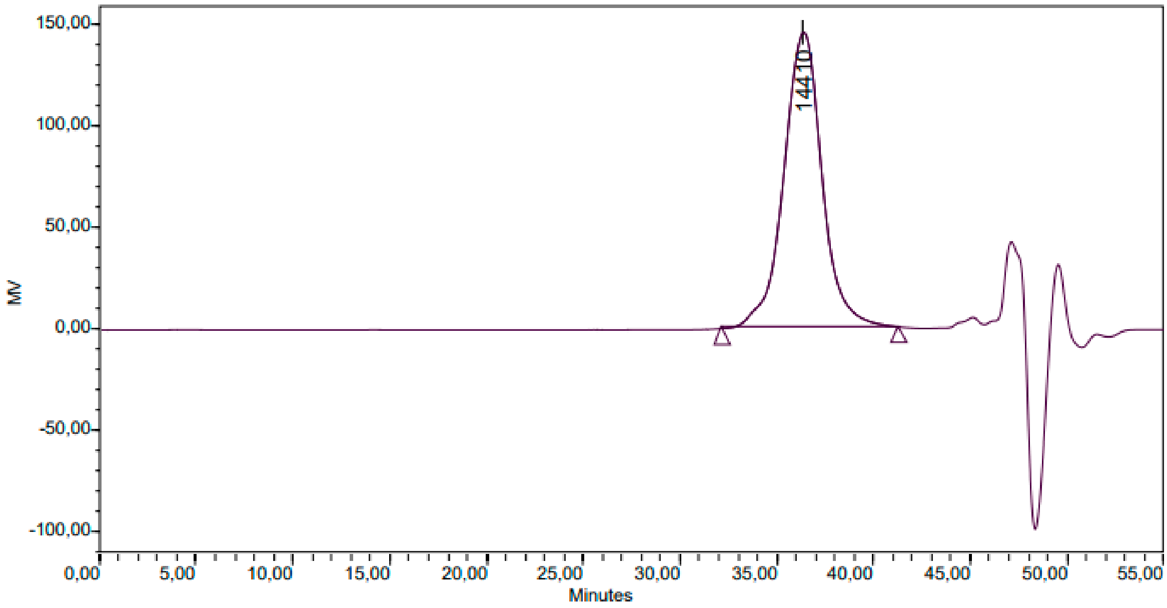

	Distribution Name	Mn (Daltons)	Mw (Daltons)	MP (Daltons)	Mz (Daltons)	Mz+1 (Daltons)	Polydispersity	Mz/Mw	Mz+1/Mw
1		13401	15719	14410	18477	22144	1,172971	1,175470	1,408711

Figure S26: SEC of PBL obtained using [NSSN-iPr]Zn (**1**) as catalyst (entry 1, Table 2).

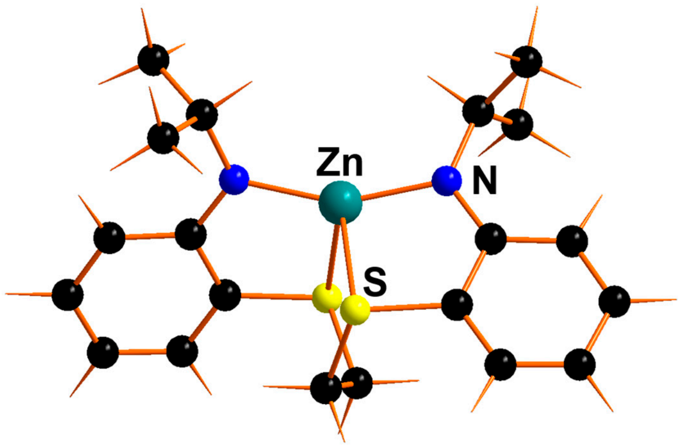

Structural features	
Zn-S	2.53 Å
Zn-N	1.94 Å
N-Zn-N	156.2°
S-Zn-S	90.6°
S-Zn-N	86.1°

	Charges	
Atom	Mulliken	APT
Zn	0.15	1.39
N	-0.10	-1.22
S	0.01	-0.09

Figure S27: Minimum-energy structure of complex **1**.

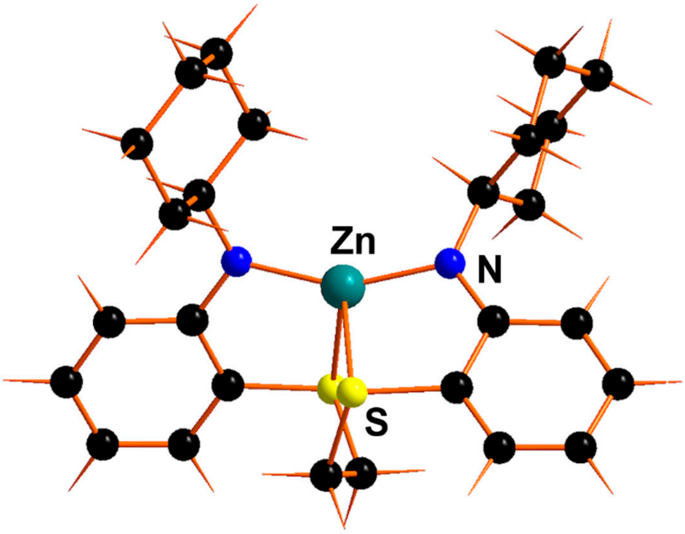

Structural features	
Zn-S	2.52 Å
Zn-N	1.93 Å
N-Zn-N	155.0°
S-Zn-S	90.1°
S-Zn-N	86.0°

	Charges	
Atom	Mulliken	APT
Zn	0.11	1.39
N	-0.06	-1.24
S	0.01	-0.09

Figure S28: Minimum-energy structure of complex **2**.

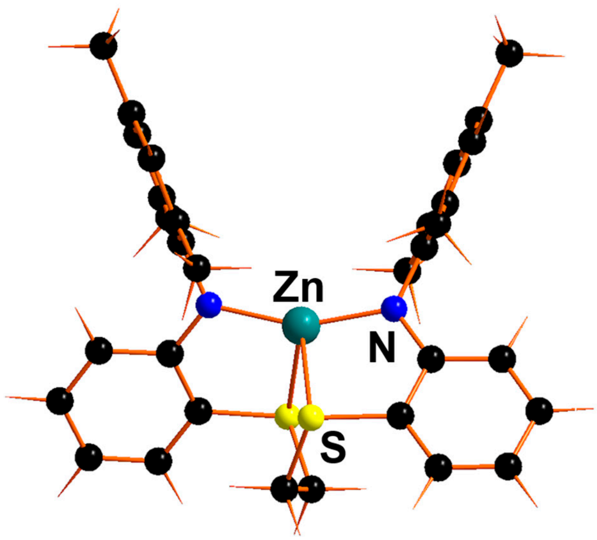

Structural features

**Zn-S**	2.56 Å
**Zn-N**	1.93 Å
**N-Zn-N**	157.4°
**S-Zn-S**	88.4°
**S-Zn-N**	85.2°

Charges

Atom	Mulliken	APT
Zn	0.18	1.23
N	-0.01	-1.26
S	0.01	-0.11

Figure S29: Minimum-energy structure of complex **3**.

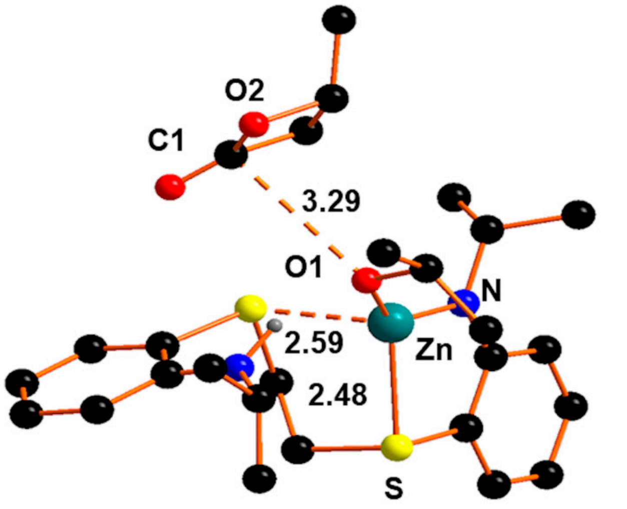

6

Figure S30: Minimum-energy structure of adduct **6**. Hydrogen atoms, except the hydrogen bonded one, were hidden for clarity.

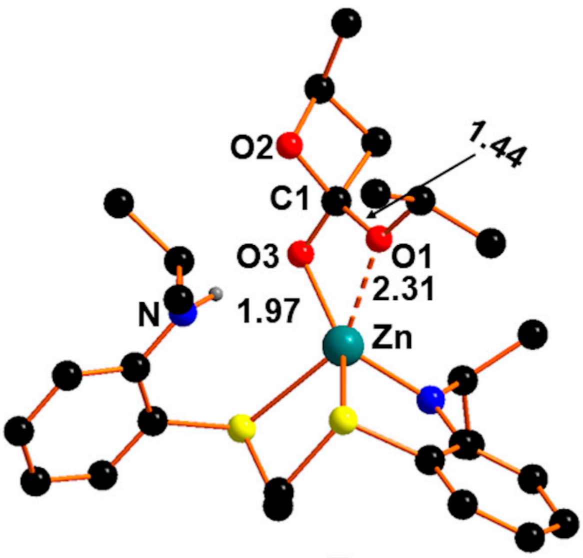

Figure S31: Minimum-energy structure of intermediate **7**. Hydrogen atoms, except the hydrogen bonded one, were hidden for clarity.

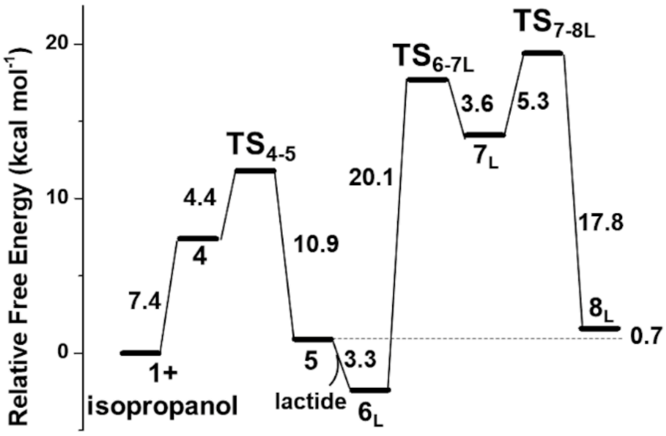

Figure S32. Relative free energy (kcal mol^{-1}) pathway for the formation of $\mathbf{8_L}$ starting from **1** together with isopropanol and LLA.

Cartesian coordinates and free energies of all the structures optimized in the computational analysis (B97D level of theory).

Compound 1
Cartesian Coordinates

Zn	-0.0001	0.6800	0.0005
S	-0.7009	-1.0979	-1.6548
N	-1.8892	1.0750	0.1760
C	-2.3742	-1.0269	-0.9819
C	-2.7732	0.0849	-0.1470
C	-3.2610	-2.0469	-1.3438
H	-2.9049	-2.8444	-1.9945
C	-4.1379	0.0332	0.2825
H	-4.5202	0.8165	0.9219
C	0.0772	-2.5329	-0.7556
H	-0.3662	-3.4529	-1.1541
H	1.1341	-2.4942	-1.0446
C	-4.5798	-2.0578	-0.8887
H	-5.2584	-2.8589	-1.1707
C	-5.0006	-0.9971	-0.0738
S	0.7014	-1.0979	1.6552
N	1.8890	1.0752	-0.1759
C	2.3745	-1.0267	0.9819
C	2.7732	0.0852	0.1468
C	3.2615	-2.0466	1.3435
H	2.9058	-2.8441	1.9943
C	4.1378	0.0336	-0.2832
H	4.5198	0.8169	-0.9226
C	-0.0768	-2.5329	0.7561
H	0.3665	-3.4529	1.1547
H	-1.1337	-2.4940	1.0451
C	4.5803	-2.0573	0.8879
H	5.2590	-2.8584	1.1697
C	5.0006	-0.9967	0.0729
C	2.2523	2.2586	-0.9860
C	2.5621	1.9361	-2.4629
C	3.3071	3.1730	-0.3307
H	1.3325	2.8598	-1.0083
H	1.7404	1.3480	-2.8916
H	2.6555	2.8704	-3.0333
H	3.4888	1.3672	-2.5898
H	3.0106	3.3998	0.7013
H	4.3087	2.7331	-0.3076
H	3.3650	4.1155	-0.8918
C	-2.2529	2.2584	0.9860
C	-2.5632	1.9359	2.4628
C	-3.3074	3.1727	0.3302
H	-1.3331	2.8597	1.0087
H	-1.7417	1.3478	2.8918
H	-2.6568	2.8703	3.0331
H	-3.4900	1.3671	2.5894
H	-3.0104	3.3996	-0.7016
H	-4.3090	2.7327	0.3066
H	-3.3657	4.1152	0.8913
H	6.0250	-0.9689	-0.2972
H	-6.0250	-0.9694	0.2959

Free Energy: -1910.729093 hartrees

Compound iso-propanol
Cartesian Coordinates

O	-0.0000	1.4286	0.0246
H	-0.0010	1.4400	0.9933
C	0.0000	0.0394	-0.3786
C	-1.2689	-0.6728	0.1003
C	1.2690	-0.6726	0.1004
H	-0.0000	0.0802	-1.4756
H	-2.1635	-0.1444	-0.2511
H	-1.2981	-1.7055	-0.2728
H	-1.2899	-0.7070	1.2000
H	2.1636	-0.1441	-0.2510
H	1.2900	-0.7068	1.2000
H	1.2984	-1.7053	-0.2727

Free Energy: -194.231097 hartrees

Compound 4
Cartesian Coordinates

Zn	-0.0007	0.3700	-0.1426
S	0.5334	-1.2380	1.7818

N	1.9684	0.3221	-0.4593
C	2.2332	-1.4233	1.2406
C	2.7456	-0.6090	0.1595
C	3.0234	-2.3648	1.9126
H	2.5758	-2.9305	2.7286
C	4.1150	-0.8862	-0.1712
H	4.5845	-0.3443	-0.9789
C	-0.3299	-2.6578	0.9250
H	-0.0316	-3.5759	1.4449
H	-1.3987	-2.4822	1.0956
C	4.3514	-2.5927	1.5536
H	4.9538	-3.3286	2.0804
C	4.8786	-1.8348	0.4975
S	-0.7237	-1.4946	-1.6809
N	-1.9603	0.5071	0.3168
C	-2.4369	-1.3916	-1.1520
C	-2.8480	-0.3917	-0.1904
C	-3.3361	-2.3046	-1.7171
H	-2.9612	-3.0194	-2.4482
C	-4.2403	-0.4551	0.1506
H	-4.6409	0.2425	0.8720
C	-0.0296	-2.8197	-0.5581
H	-0.4453	-3.7746	-0.9004
H	1.0506	-2.8118	-0.7466
C	-4.6829	-2.3202	-1.3549
H	-5.3711	-3.0360	-1.7978
C	-5.1144	-1.3795	-0.4084
C	-2.3132	1.4136	1.4290
C	-2.6748	0.6941	2.7449
C	-3.3313	2.5285	1.0806
H	-1.3800	1.9473	1.6495
H	-1.8662	0.0090	3.0258
H	-2.7984	1.4337	3.5481
H	-3.6019	0.1175	2.6667
H	-3.1365	2.9470	0.0826
H	-4.3706	2.1861	1.0857
H	-3.2448	3.3388	1.8172
C	2.4529	1.0990	-1.6206
C	2.7231	0.2453	-2.8782
C	3.6030	2.0853	-1.3108
H	1.6034	1.7334	-1.8900
H	1.8309	-0.3461	-3.1191
H	2.9421	0.9044	-3.7299
H	3.5654	-0.4431	-2.7556
H	3.4039	2.6078	-0.3677
H	4.5849	1.6090	-1.2309
H	3.6614	2.8307	-2.1162
H	-6.1590	-1.3602	-0.0986
H	5.9116	-1.9842	0.1840
O	-0.2175	2.6458	-0.7214
H	-1.1289	2.7756	-0.4187
C	0.6245	3.7137	-0.1841
C	1.0902	3.3739	1.2296
C	-0.1086	5.0503	-0.2626
H	1.4893	3.7267	-0.8546
H	1.5797	2.3930	1.2376
H	1.8079	4.1284	1.5784
H	0.2399	3.3624	1.9250
H	-0.4278	5.2610	-1.2905
H	-0.9934	5.0393	0.3904
H	0.5513	5.8593	0.0747

Free Energy: -2104.948385 hartrees

Transition State TS_{4-5}
Imaginary frequency at -1054 cm^{-1}
Cartesian Coordinates

Zn	0.1796	0.4574	0.0141
S	0.8541	-1.5909	1.4330
N	2.1266	0.7834	-0.0398
C	2.5613	-1.4345	0.8900
C	2.9958	-0.2326	0.2139
C	3.4339	-2.4887	1.1835
H	3.0463	-3.3559	1.7166
C	4.3846	-0.2370	-0.1444
H	4.7967	0.6136	-0.6690
C	0.1655	-2.8568	0.2542
H	0.6504	-3.8130	0.4827
H	-0.8985	-2.9305	0.5077
C	4.7764	-2.4473	0.8064
H	5.4445	-3.2740	1.0345
C	5.2329	-1.3004	0.1398
S	-0.5230	-1.0340	-1.8423
N	-2.0009	0.3240	0.5369
C	-2.2009	-1.3441	-1.2502
C	-2.7324	-0.6710	-0.1020
C	-2.9406	-2.3092	-1.9473
H	-2.4997	-2.7772	-2.8257
C	-4.0312	-1.0888	0.2965
H	-4.4928	-0.6240	1.1564
C	0.3593	-2.5543	-1.2247
H	0.0023	-3.4054	-1.8159
H	1.4162	-2.3755	-1.4555

C	-4.2129	-2.6893	-1.5222	H	3.8410	1.3225	-2.3243
H	-4.7740	-3.4445	-2.0672	H	3.1907	2.9901	1.1585
C	-4.7465	-2.0681	-0.3869	H	4.5513	2.4385	0.1634
C	-2.3264	0.7830	1.9120	H	3.6350	3.8778	-0.3204
C	-2.3800	-0.3391	2.9598	H	-5.7393	-2.3395	-0.0311
C	-3.5479	1.7223	2.0003	H	6.2759	-1.2303	-0.1680
H	-1.4623	1.4068	2.1762	O	-0.9311	2.0641	-0.7971
H	-1.4630	-0.9375	2.9290	H	-1.7211	1.3620	-0.1706
H	-2.4683	0.1069	3.9592	C	-0.8619	3.4718	-0.5796
H	-3.2340	-1.0083	2.8123	C	-0.5383	3.8101	0.8816
H	-4.5060	1.1964	1.9339	C	-2.1612	4.1406	-1.0435
H	-3.5256	2.2519	2.9625	H	-0.0407	3.8555	-1.2086
H	-3.5115	2.4678	1.1980	H	0.3801	3.2976	1.1974
C	2.5366	2.0463	-0.6855	H	-0.3917	4.8919	1.0066
C	2.9197	1.8944	-2.1724	H	-1.3603	3.4960	1.5374
C	3.5498	2.8781	0.1273	H	-2.3643	3.8873	-2.0913
H	1.6231	2.6484	-0.6913	H	-3.0071	3.7981	-0.4325
H	2.1082	1.3833	-2.7063	H	-2.0884	5.2330	-0.9509
H	3.0571	2.8879	-2.6215				

Free energy : -2104.941390 hartrees

Compound 5

Cartesian Coordinates

Zn	0.6265	0.8560	-0.3662	C	-4.0474	-3.2085	-1.5110
S	0.6941	-0.5250	1.7316	H	-4.4105	-4.1112	-1.9959
N	2.4914	0.3534	-0.6293	C	-4.8190	-2.5363	-0.5551
C	2.4521	-0.8570	1.4933	C	-3.2945	1.1567	1.3439
C	3.1444	-0.3748	0.3199	C	-3.4235	0.4016	2.6765
C	3.0981	-1.5863	2.4981	C	-4.6094	1.8351	0.9083
H	2.5221	-1.9046	3.3658	H	-2.5730	1.9658	1.5106
C	4.5287	-0.7384	0.2675	H	-2.4447	0.0147	2.9857
H	5.1254	-0.4267	-0.5782	H	-3.7877	1.0893	3.4507
C	-0.0570	-2.0829	1.0409	H	-4.1232	-0.4390	2.6156
H	0.3307	-2.9079	1.6498	H	-4.4681	2.3441	-0.0525
H	-1.1359	-1.9899	1.2067	H	-5.4389	1.1275	0.8045
C	4.4510	-1.9133	2.4016	H	-4.8969	2.5835	1.6590
H	4.9427	-2.4826	3.1864	C	3.1390	0.8400	-1.8675
C	5.1505	-1.4770	1.2677	C	3.5842	-0.2872	-2.8218
S	-0.5673	-1.1389	-1.5835	C	4.2271	1.9082	-1.6330
N	-2.6437	0.3595	0.2961	H	2.3387	1.3662	-2.4058
C	-2.2970	-1.5336	-1.2092	H	2.7413	-0.9646	-3.0092
C	-3.0972	-0.7979	-0.2755	H	3.8980	0.1487	-3.7799
C	-2.7908	-2.6916	-1.8269	H	4.4172	-0.8798	-2.4307
H	-2.1607	-3.2069	-2.5499	H	3.8246	2.7104	-1.0011
C	-4.3609	-1.3676	0.0434	H	5.1251	1.5126	-1.1482
H	-4.9977	-0.8664	0.7598	H	4.5250	2.3422	-2.5972
C	0.2578	-2.3380	-0.4275	H	-5.8000	-2.9185	-0.2771
H	-0.0821	-3.3416	-0.7071	H	6.2088	-1.7120	1.1585
H	1.3318	-2.2494	-0.6253	O	-0.6928	2.1496	-0.7114

H	-1.9604	0.9251	-0.2212
C	-0.5309	3.5149	-0.3451
C	-0.0315	3.6718	1.0997
C	-1.8674	4.2397	-0.5438
H	0.2171	3.9879	-1.0116
H	0.9395	3.1715	1.2297
H	0.0924	4.7319	1.3629
H	-0.7501	3.2187	1.7973
H	-2.2227	4.1052	-1.5733
H	-2.6217	3.8301	0.1410
H	-1.7605	5.3152	-0.3448

Free energy : -2104.958799 hartrees

Compound 6

Cartesian Coordinates

Zn	0.7012	0.3394	0.4647
S	1.8256	-1.7639	1.1334
N	2.4478	0.9626	-0.1705
C	3.4279	-1.1409	0.5802
C	3.5418	0.1665	-0.0264
C	4.5316	-1.9799	0.7698
H	4.3799	-2.9458	1.2493
C	4.8706	0.5040	-0.4420
H	5.0482	1.4578	-0.9192
C	1.3040	-2.7711	-0.3465
H	2.0458	-3.5714	-0.4491
H	0.3353	-3.2078	-0.0783
C	5.8080	-1.6021	0.3515
H	6.6593	-2.2613	0.5010
C	5.9537	-0.3478	-0.2587
S	-0.2026	-0.7810	-1.6969
N	-1.9520	-1.3044	0.7643
C	-1.5723	-1.9473	-1.5387
C	-2.2528	-2.1194	-0.3020
C	-1.8855	-2.7183	-2.6638
H	-1.3620	-2.5258	-3.5987
C	-3.1998	-3.1772	-0.2540
H	-3.7407	-3.3611	0.6640
C	1.2139	-1.9853	-1.6490
H	1.0740	-2.6812	-2.4834
H	2.1171	-1.3897	-1.8218
C	-2.8344	-3.7396	-2.5942
H	-3.0668	-4.3370	-3.4722
C	-3.4784	-3.9654	-1.3681
C	-2.4333	-1.3953	2.1504
C	-2.0165	-2.7016	2.8455
C	-3.9308	-1.0691	2.3230
H	-1.8813	-0.5871	2.6477
H	-0.9327	-2.8464	2.7550
H	-2.2737	-2.6423	3.9108
H	-2.5171	-3.5808	2.4268
H	-4.1707	-0.1311	1.8079
H	-4.5831	-1.8545	1.9269
H	-4.1564	-0.9488	3.3912
C	2.4941	2.3145	-0.7637
C	2.8266	2.3233	-2.2697
C	3.3161	3.3351	0.0501
H	1.4575	2.6770	-0.7004
H	2.1481	1.6427	-2.7999
H	2.6860	3.3363	-2.6713
H	3.8548	2.0140	-2.4831
H	2.9872	3.3205	1.0971
H	4.3924	3.1381	0.0293
H	3.1477	4.3429	-0.3535
H	-4.2221	-4.7557	-1.2789
H	6.9356	-0.0212	-0.5996
O	-0.8879	1.1481	1.0819
H	-1.6030	-0.3656	0.5604
C	-0.9260	2.0153	2.2045
C	-0.0429	1.5221	3.3616
C	-2.3805	2.1703	2.6666
H	-0.5516	3.0172	1.9068
H	1.0124	1.4674	3.0561
H	-0.1113	2.1980	4.2260
H	-0.3688	0.5194	3.6738
H	-3.0218	2.4681	1.8298
H	-2.7515	1.2137	3.0612
H	-2.4544	2.9265	3.4609
C	-2.8516	2.0406	-1.4146
C	-1.5950	2.8920	-1.5342
C	-2.3561	3.8773	-0.6276
H	-0.7232	2.4187	-1.0794
H	-1.9635	3.9262	0.3914
O	-3.5327	2.9373	-0.6208
O	-3.2489	0.9759	-1.8029
C	-2.7230	5.2325	-1.1852
H	-3.4662	5.7282	-0.5486
H	-1.8240	5.8626	-1.2246
H	-3.1288	5.1353	-2.2003
H	-1.3813	3.2436	-2.5494

Free Energy: -2411.273339 hartrees

Transition State $TS_{6\text{-}7}$
Imaginary Frequency at -173 cm^{-1}
Cartesian Coordinates

Zn	0.1790	-0.0071	0.2706
S	1.0190	-2.3125	0.9774
N	2.1512	0.3769	-0.0151
C	2.7364	-1.8896	0.6772
C	3.0896	-0.5873	0.1634
C	3.6868	-2.8837	0.9413
H	3.3492	-3.8341	1.3525
C	4.4891	-0.4440	-0.1248
H	4.8453	0.4770	-0.5621
C	0.6129	-3.1981	-0.6085
H	1.3150	-4.0368	-0.6775
H	-0.4010	-3.5924	-0.4829
C	5.0405	-2.6803	0.6760
H	5.7722	-3.4567	0.8845
C	5.4183	-1.4450	0.1277
S	-0.4857	-0.9445	-1.9761
N	-2.0550	-0.5419	0.6237
C	-1.9864	-1.8062	-1.4720
C	-2.5691	-1.5826	-0.1974
C	-2.4995	-2.7737	-2.3466
H	-2.0376	-2.9066	-3.3226
C	-3.6578	-2.4041	0.1560
H	-4.1459	-2.2626	1.1098
C	0.7335	-2.3553	-1.8706
H	0.5804	-2.9904	-2.7503
H	1.7164	-1.8756	-1.9366
C	-3.5755	-3.5749	-1.9678
H	-3.9646	-4.3271	-2.6498
C	-4.1458	-3.3867	-0.7051
C	-2.4193	-0.4265	2.0743
C	-2.1239	-1.6813	2.8997
C	-3.8284	0.1443	2.3077
H	-1.7260	0.3456	2.4199
H	-1.0920	-2.0146	2.7501
H	-2.2541	-1.4332	3.9605
H	-2.7922	-2.5166	2.6684
H	-3.9717	1.0301	1.6793
H	-4.6278	-0.5737	2.0952
H	-3.9185	0.4424	3.3609
C	2.5005	1.7274	-0.5043
C	2.8735	1.7546	-2.0003
C	3.5164	2.5314	0.3456
H	1.5657	2.2834	-0.4084
H	2.1061	1.2422	-2.5943
H	2.9415	2.7944	-2.3489
H	3.8340	1.2653	-2.1968
H	3.3708	2.3421	1.4146
H	4.5625	2.3181	0.1034
H	3.3499	3.6016	0.1625
H	-4.9909	-3.9951	-0.3895
H	6.4641	-1.2554	-0.1130
O	-0.5140	1.9300	0.6221
H	-2.3318	0.3881	0.1902
C	-0.2251	2.7433	1.7936
C	0.7019	1.9462	2.7100
C	-1.4883	3.1750	2.5458
H	0.2967	3.6423	1.4363
H	1.5741	1.5706	2.1677
H	1.0394	2.5862	3.5352
H	0.1656	1.0900	3.1446
H	-2.1791	3.7070	1.8893
H	-2.0041	2.3106	2.9797
H	-1.1917	3.8441	3.3659
C	-1.6003	2.5323	-0.5677
C	-0.7733	2.5892	-1.8910
C	-0.5359	4.0635	-1.5468
H	0.0997	1.9439	-1.9833
H	0.4680	4.2421	-1.1279
O	-1.5235	4.0078	-0.4636
O	-2.7244	1.9677	-0.4371
C	-0.8790	5.1126	-2.5870
H	-0.8691	6.1183	-2.1459
H	-0.1422	5.0850	-3.4025
H	-1.8758	4.9183	-3.0033
H	-1.4370	2.4560	-2.7525

Free Energy: -2411.239149 hartrees

Compound 7
Cartesian Coordinates

Zn	0.4726	0.0218	-0.5687
S	0.5561	-1.1860	1.5933
N	2.3303	-0.5586	-0.8499
C	2.3202	-1.5093	1.3944
C	3.0049	-1.1417	0.1739
C	2.9813	-2.0947	2.4802
H	2.4091	-2.3307	3.3763
C	4.4046	-1.4522	0.1746
H	4.9983	-1.2126	-0.6966
C	-0.1897	-2.7930	1.0225
H	0.2493	-3.5770	1.6500
H	-1.2604	-2.7154	1.2436
C	4.3463	-2.3799	2.4292
H	4.8505	-2.8383	3.2762
C	5.0414	-2.0479	1.2568
S	-0.8298	-1.9682	-1.6173
N	-2.6437	-0.0672	0.1343
C	-2.5297	-2.2072	-1.0370
C	-3.2007	-1.2809	-0.1769
C	-3.1286	-3.4237	-1.3937
H	-2.5983	-4.0902	-2.0717
C	-4.4466	-1.7099	0.3542
H	-4.9881	-1.0500	1.0192
C	0.0442	-3.1234	-0.4466
H	-0.3167	-4.1366	-0.6561
H	1.1050	-3.0487	-0.7096
C	-4.3640	-3.8064	-0.8709
H	-4.8108	-4.7567	-1.1525
C	-5.0075	-2.9380	0.0199
C	-3.0783	0.8877	1.1657
C	-3.0799	0.2776	2.5751
C	-4.3861	1.6293	0.8288
H	-2.2883	1.6434	1.1477
H	-2.1062	-0.1790	2.7904
H	-3.2601	1.0735	3.3090
H	-3.8563	-0.4841	2.7064
H	-4.3209	2.0563	-0.1787
H	-5.2684	0.9811	0.8788
H	-4.5284	2.4518	1.5426
C	2.9692	-0.1355	-2.1117
C	3.5014	-1.3029	-2.9682
C	3.9871	1.0114	-1.9418
H	2.1491	0.2971	-2.7012
H	2.7088	-2.0501	-3.1038
H	3.7946	-0.9256	-3.9574
H	4.3689	-1.8034	-2.5265
H	3.5150	1.8396	-1.3985
H	4.8838	0.7103	-1.3908
H	4.2994	1.3759	-2.9301
H	-5.9702	-3.2107	0.4494
H	6.1093	-2.2518	1.1836
O	0.5026	2.1459	0.3345
H	-2.0526	0.3715	-0.5706
C	0.9390	3.0767	1.3560
C	2.3502	2.6567	1.7591
C	-0.0419	3.0580	2.5274
H	0.9648	4.0810	0.9136
H	3.0280	2.6992	0.8985
H	2.7296	3.3208	2.5464
H	2.3425	1.6288	2.1443
H	-1.0408	3.3460	2.1851
H	-0.0863	2.0495	2.9591
H	0.2883	3.7584	3.3066
C	-0.2982	2.5626	-0.7989
C	0.2432	3.7508	-1.6412
C	-1.0672	4.4674	-1.2690
H	1.1432	4.2248	-1.2347
H	-1.7779	4.5231	-2.1061
O	-1.4539	3.3671	-0.3725
O	-0.6114	1.4225	-1.4255
H	0.3847	3.5026	-2.6964
C	-0.9826	5.8028	-0.5536
H	-0.2534	5.7609	0.2655
H	-0.6725	6.5845	-1.2612
H	-1.9604	6.0808	-0.1386

Free Energy: -2411. 260720 hartrees

Transition State TS_{7-8}
Imaginary Frequency at -102 cm^{-1}
Cartesian Coordinates

Zn	0.6374	0.0177	-0.1426
S	-0.0926	-1.4084	1.7406
N	1.8801	-1.4233	-0.6359
C	1.2958	-2.5287	1.4580
C	2.1187	-2.4030	0.2739
C	1.5208	-3.5094	2.4303
H	0.8730	-3.5380	3.3053
C	3.1527	-3.3916	0.1768
H	3.8138	-3.3801	-0.6786
C	-1.4815	-2.3635	0.9481
H	-1.5720	-3.2931	1.5223
H	-2.3792	-1.7547	1.0980

C	2.5466	-4.4452	2.2911
H	2.7130	-5.2047	3.0507
C	3.3530	-4.3673	1.1459
S	-1.3811	-1.2166	-1.6442
N	-2.6931	0.8460	0.3082
C	-3.1359	-0.8290	-1.4059
C	-3.5949	0.1167	-0.4361
C	-4.0467	-1.5617	-2.1779
H	-3.6593	-2.2583	-2.9192
C	-4.9991	0.2161	-0.2645
H	-5.3974	0.9179	0.4562
C	-1.2670	-2.6894	-0.5253
H	-2.0285	-3.4072	-0.8516
H	-0.2729	-3.1156	-0.6955
C	-5.4254	-1.4351	-1.9947
H	-6.1172	-2.0174	-2.5985
C	-5.8883	-0.5433	-1.0213
C	-2.9951	1.7664	1.4120
C	-3.6613	1.0687	2.6083
C	-3.7203	3.0577	0.9831
H	-2.0006	2.0692	1.7580
H	-3.0678	0.1981	2.9134
H	-3.7106	1.7695	3.4514
H	-4.6798	0.7319	2.3907
H	-3.1899	3.5235	0.1431
H	-4.7550	2.8748	0.6742
H	-3.7363	3.7664	1.8222
C	2.6611	-1.2558	-1.8774
C	2.5003	-2.4189	-2.8773
C	4.1326	-0.8530	-1.6457
H	2.2019	-0.3898	-2.3721
H	1.4340	-2.6005	-3.0626
H	2.9766	-2.1499	-3.8299
H	2.9501	-3.3530	-2.5255
H	4.1749	-0.0104	-0.9445
H	4.7427	-1.6654	-1.2379
H	4.5800	-0.5367	-2.5979
H	-6.9574	-0.4219	-0.8545
H	4.1635	-5.0818	1.0040
O	1.6461	3.0766	-1.2097
H	-1.8299	1.0954	-0.1719
C	2.7635	3.8985	-0.7446
C	2.4843	5.3599	-1.0855
C	4.0187	3.3617	-1.4237
H	2.8631	3.7685	0.3391
H	1.5665	5.7105	-0.5982
H	3.3206	5.9885	-0.7525
H	2.3694	5.4758	-2.1709
H	4.1805	2.3129	-1.1516
H	3.9201	3.4348	-2.5145
H	4.8914	3.9495	-1.1104
C	0.7588	2.5737	-0.2939
C	0.3248	3.4142	0.9379
C	0.8693	2.3334	1.8847
H	0.8603	4.3634	1.0357
H	0.0745	1.6982	2.3022
O	1.5780	1.6023	0.8220
O	-0.1176	1.7750	-0.8501
H	-0.7528	3.5976	0.9545
C	1.8129	2.7819	2.9843
H	2.6096	3.4138	2.5705
H	1.2621	3.3603	3.7393
H	2.2708	1.9139	3.4751

Free Energy: -2411.253205 hartrees

Compound 8

Cartesian Coordinates

Zn	-0.8218	-0.6440	0.5138
S	-1.9342	1.6174	1.1635
N	-2.6278	-1.1836	-0.0155
C	-3.5333	1.0166	0.5914
C	-3.6788	-0.3146	0.0492
C	-4.6110	1.9049	0.6936
H	-4.4353	2.8864	1.1318
C	-5.0060	-0.6130	-0.4054
H	-5.2086	-1.5766	-0.8495
C	-1.4462	2.6772	-0.2848
H	-2.1987	3.4710	-0.3642
H	-0.4807	3.1179	-0.0173
C	-5.8869	1.5625	0.2460
H	-6.7142	2.2626	0.3314
C	-6.0606	0.2868	-0.3082
S	0.0065	0.6811	-1.6962
N	1.7243	1.9167	0.6783
C	1.4402	1.7880	-1.7468
C	2.1001	2.2962	-0.5800
C	1.8635	2.1640	-3.0297
H	1.3484	1.7409	-3.8900
C	3.1400	3.2386	-0.8094
H	3.6560	3.6706	0.0375
C	-1.3694	1.9186	-1.6037
H	-1.2258	2.6226	-2.4318
H	-2.2889	1.3465	-1.7765

C	2.9037	3.0746	-3.2219
H	3.2120	3.3564	-4.2256
C	3.5283	3.6139	-2.0913
C	2.2735	2.3629	1.9659
C	1.9904	3.8471	2.2553
C	3.7508	1.9847	2.1937
H	1.6883	1.7805	2.6864
H	0.9164	4.0500	2.1628
H	2.3026	4.0845	3.2807
H	2.5275	4.5169	1.5748
H	3.9072	0.9232	1.9690
H	4.4401	2.5721	1.5779
H	4.0087	2.1572	3.2469
C	-2.7808	-2.5719	-0.5087
C	-3.0501	-2.6698	-2.0249
C	-3.7173	-3.4526	0.3462
H	-1.7909	-3.0162	-0.3761
H	-2.2771	-2.1100	-2.5664
H	-2.9973	-3.7218	-2.3382
H	-4.0275	-2.2763	-2.3225
H	-3.4303	-3.3731	1.4030
H	-4.7763	-3.1900	0.2636
H	-3.6040	-4.5003	0.0349
H	4.3367	4.3348	-2.2041
H	-7.0399	-0.0191	-0.6754
O	2.4126	-3.1493	-0.8844
H	1.1502	1.0796	0.7854
C	3.7253	-3.6624	-0.4400
C	4.6325	-2.5031	-0.0433
C	4.2651	-4.4548	-1.6230
H	3.5573	-4.3382	0.4073
H	4.2377	-1.9574	0.8196
H	5.6249	-2.8928	0.2161
H	4.7369	-1.8062	-0.8841
H	3.5755	-5.2605	-1.8993
H	4.4102	-3.7952	-2.4873
H	5.2322	-4.8948	-1.3501
C	1.4511	-2.7316	-0.0415
C	1.6537	-2.6751	1.4463
C	1.7630	-1.1804	1.9246
H	0.7652	-3.1021	1.9266
H	2.5544	-0.7042	1.3101
O	0.5737	-0.4663	1.8056
O	0.4037	-2.3401	-0.5565
H	2.5364	-3.2247	1.7800
C	2.2328	-1.1797	3.3868
H	1.5067	-1.7169	4.0115
H	3.2171	-1.6584	3.4884
H	2.3057	-0.1469	3.7457

Free Energy: -2411.294356 hartrees

Compound rac-β-butyrolactone

Cartesian Coordinates

C	1.076034	-0.008436	-0.038350
C	0.099427	1.157810	0.091815
C	-0.918901	0.066981	0.481410
H	0.366844	1.880641	0.869138
H	-1.208848	0.078370	1.536734
O	0.156691	-0.974623	0.306283
O	2.231256	-0.174844	-0.318174
C	-2.089657	-0.175219	-0.442344
H	-2.583130	-1.125399	-0.204937
H	-2.819810	0.636199	-0.320583
H	-1.756890	-0.195427	-1.487835
H	-0.103152	1.674530	-0.852577

Free Energy: -306.328048 hartrees

Compound L-lactide

Cartesian Coordinates

C	2.303424	-0.901691	-1.453030
C	2.674411	-2.068754	-2.368437
C	4.690590	-0.609171	-1.015688
H	1.911827	-2.835258	-2.206406
H	5.179102	-0.518445	-0.041768
O	3.285448	-0.311483	-0.735549
O	1.159845	-0.519953	-1.356091
C	2.682791	-1.655165	-3.842000
H	3.445650	-0.894517	-4.039678
H	2.887537	-2.534918	-4.461146
H	1.699756	-1.249594	-4.105396
C	4.955333	-2.038193	-1.490652
O	3.928464	-2.738832	-2.021731
O	6.049696	-2.541716	-1.382846
C	5.271126	0.427546	-1.980455
H	5.116268	1.429682	-1.566982
H	6.345511	0.246898	-2.095616
H	4.793519	0.369966	-2.964429

Free Energy: -534.114769 hartrees

Compound 6_L

Cartesian Coordinates

Zn	1.0261	0.2229	0.6232
S	2.1963	-1.9380	1.0070
N	2.6812	0.8928	-0.1460
C	3.7439	-1.2415	0.3893
C	3.7967	0.1102	-0.1206
C	4.8743	-2.0651	0.4473
H	4.7712	-3.0681	0.8585
C	5.0914	0.5140	-0.5784
H	5.2234	1.5109	-0.9753
C	1.5561	-2.8265	-0.5028
H	2.2782	-3.6214	-0.7234
H	0.6050	-3.2727	-0.1934
C	6.1148	-1.6254	-0.0150
H	6.9861	-2.2736	0.0310
C	6.2000	-0.3231	-0.5278
S	0.0181	-0.6926	-1.5825
N	-1.5689	-1.9531	0.8662
C	-1.4215	-1.7898	-1.5687
C	-2.0026	-2.3356	-0.3796
C	-1.9236	-2.1212	-2.8352
H	-1.4841	-1.6484	-3.7106
C	-3.0220	-3.3045	-0.5742
H	-3.4776	-3.7718	0.2879
C	1.3862	-1.9384	-1.7282
H	1.1599	-2.5550	-2.6053
H	2.2924	-1.3534	-1.9212
C	-2.9570	-3.0444	-2.9888
H	-3.3339	-3.2896	-3.9783
C	-3.4853	-3.6460	-1.8405
C	-2.0482	-2.4534	2.1644
C	-1.6889	-3.9285	2.4007
C	-3.5270	-2.1371	2.4682
H	-1.4560	-1.8684	2.8794
H	-0.6124	-4.0819	2.2564
H	-1.9457	-4.2044	3.4314
H	-2.2261	-4.6046	1.7271
H	-3.7334	-1.0803	2.2655
H	-4.2225	-2.7446	1.8804
H	-3.7264	-2.3297	3.5307
C	2.6499	2.2519	-0.7271
C	2.9320	2.2937	-2.2422

C	3.4503	3.2981	0.0740
H	1.6017	2.5630	-0.6320
H	2.2742	1.5837	-2.7589
H	2.7187	3.3014	-2.6234
H	3.9681	2.0484	-2.4950
H	3.1464	3.2667	1.1283
H	4.5322	3.1393	0.0273
H	3.2370	4.3016	-0.3188
H	-4.2830	-4.3818	-1.9265
H	7.1543	0.0553	-0.8926
O	-0.5962	0.6704	1.4733
H	-1.2250	-0.9930	0.9623
C	-0.6814	1.3852	2.7064
C	0.3985	0.9446	3.7025
C	-2.0807	1.1931	3.2992
H	-0.5386	2.4647	2.5050
H	1.4056	1.1360	3.3047
H	0.3004	1.4897	4.6516
H	0.2999	-0.1314	3.9043
H	-2.8520	1.4624	2.5692
H	-2.2229	0.1443	3.5892
H	-2.2091	1.8235	4.1891
O	-2.6196	1.4438	-3.0478
C	-2.4262	1.8570	-1.9287
C	-3.0544	1.2880	-0.6523
C	-1.3210	3.2918	-0.3240
C	-2.6368	3.3956	0.4548
O	-1.5900	2.9087	-1.7186
O	-3.5225	2.4002	0.1954
O	-2.9036	4.2641	1.2528
H	-0.7535	2.4745	0.1464
H	-2.2623	0.7773	-0.0846
C	-4.2303	0.3695	-0.9071
H	-3.8934	-0.4780	-1.5109
H	-4.6148	-0.0038	0.0475
H	-5.0243	0.8989	-1.4447
C	-0.5246	4.5807	-0.3491
H	-0.2689	4.8647	0.6767
H	0.3975	4.4311	-0.9202
H	-1.1093	5.3880	-0.8029

Free Energy: -2639.078851 hartrees

Transition State $TS_{6\text{-}7L}$

Imaginary frequency at -65.4 cm^{-1}

Cartesian Coordinates

Zn	-0.5525	-0.2657	-0.2365
S	-1.9624	0.5235	1.6787
N	-2.0769	-1.4239	-0.7144
C	-3.2357	-0.6973	1.3033
C	-3.1467	-1.5157	0.1136
C	-4.3037	-0.7930	2.2032
H	-4.3008	-0.1598	3.0893
C	-4.2686	-2.3881	-0.0801
H	-4.2945	-3.0217	-0.9556
C	-2.6701	2.0438	0.8677
H	-3.6351	2.2312	1.3522
H	-1.9808	2.8609	1.1120
C	-5.3630	-1.6721	1.9779
H	-6.1893	-1.7380	2.6812
C	-5.3268	-2.4600	0.8171
S	-1.2915	1.8123	-1.6048
N	1.3625	2.3364	-0.0089
C	-0.5343	3.3883	-1.1360
C	0.6589	3.4630	-0.3557
C	-1.2081	4.5516	-1.5347
H	-2.0946	4.4591	-2.1595
C	1.0645	4.7612	0.0522
H	1.9685	4.8670	0.6379
C	-2.8726	1.9234	-0.6369
H	-3.4234	2.7959	-1.0044
H	-3.4269	1.0138	-0.8919
C	-0.7838	5.8134	-1.1199
H	-1.3243	6.7046	-1.4292
C	0.3577	5.9020	-0.3120
C	2.3921	2.2052	1.0323
C	1.9251	2.7213	2.4019
C	3.7708	2.7624	0.6267
H	2.5029	1.1231	1.1251
H	0.9164	2.3522	2.6220
H	2.6070	2.3457	3.1751
H	1.9176	3.8146	2.4628
H	4.0336	2.4164	-0.3797
H	3.8054	3.8584	0.6411
H	4.5307	2.3945	1.3297
C	-1.9495	-2.2072	-1.9594
C	-3.0524	-1.9329	-3.0140
C	-1.7472	-3.7198	-1.7074
H	-1.0130	-1.8483	-2.4034
H	-3.4700	-0.9277	-2.8780
H	-2.6123	-1.9883	-4.0184
H	-3.8753	-2.6560	-2.9769
H	-1.7624	-4.2709	-2.6578
H	-0.7791	-3.8996	-1.2237
H	-2.5270	-4.1329	-1.0594
H	0.7170	6.8736	0.0226
H	-6.1425	-3.1511	0.6060
O	1.1962	-0.9109	0.7317
H	1.3161	1.5455	-0.6396
C	1.2551	-1.7728	1.9038
C	0.0627	-2.7301	1.9314
C	1.3056	-0.8760	3.1399
H	2.1764	-2.3585	1.8637
H	0.0004	-3.3084	1.0036
H	0.1634	-3.4303	2.7713
H	-0.8780	-2.1779	2.0521
H	2.1993	-0.2421	3.1212
H	0.4195	-0.2326	3.1832
H	1.3306	-1.4967	4.0454
C	1.9310	-1.1413	-0.7262
C	2.1235	-2.6442	-0.9974
C	4.2555	-1.0542	0.0458
H	1.1931	-3.1640	-0.7637
H	4.3053	-0.5784	1.0362
O	3.1552	-0.4658	-0.6738
O	1.0994	-0.5499	-1.5170
C	2.5278	-2.8901	-2.4474
H	3.5003	-2.4293	-2.6553
H	2.5915	-3.9679	-2.6342
H	1.7786	-2.4461	-3.1106
C	4.1705	-2.5592	0.3397
O	3.1142	-3.2705	-0.1102
O	5.0114	-3.1059	1.0208
C	5.5368	-0.7262	-0.7271
H	5.6434	0.3616	-0.7981
H	6.4036	-1.1423	-0.2036
H	5.4866	-1.1453	-1.7386

Free Energy -2639.047489 hartrees

Compound 7_L

Cartesian Coordinates

Zn	-0.5091	-0.2833	-0.3578
S	-1.8485	0.2205	1.6561
N	-1.7853	-1.7403	-0.7219
C	-2.8791	-1.2387	1.3945
C	-2.7299	-2.0445	0.2014
C	-3.8093	-1.5415	2.3957
H	-3.8567	-0.9047	3.2779
C	-3.6487	-3.1418	0.1205
H	-3.6224	-3.7872	-0.7468
C	-2.9119	1.5615	0.9213
H	-3.8518	1.5511	1.4847
H	-2.3836	2.5008	1.1219
C	-4.6725	-2.6305	2.2743
H	-5.3939	-2.8571	3.0552
C	-4.5772	-3.4179	1.1166
S	-1.7100	1.6128	-1.6509
N	0.7837	2.5288	0.0254
C	-1.2241	3.2815	-1.1453
C	-0.0911	3.5360	-0.3131
C	-2.0824	4.3199	-1.5333
H	-2.9178	4.0929	-2.1932
C	0.0508	4.8649	0.1644
H	0.8869	5.1064	0.8071
C	-3.2068	1.4014	-0.5648
H	-3.9448	2.1499	-0.8733
H	-3.5862	0.3993	-0.7916
C	-1.9059	5.6202	-1.0610
H	-2.5853	6.4129	-1.3642
C	-0.8357	5.8754	-0.1945
C	1.8219	2.5602	1.0690
C	1.2485	2.8758	2.4576
C	3.0506	3.4175	0.7108
H	2.1628	1.5228	1.0989
H	0.4179	2.1964	2.6838
H	2.0329	2.7278	3.2105
H	0.8873	3.9061	2.5452
H	3.4247	3.1328	-0.2794
H	2.8364	4.4920	0.7070
H	3.8456	3.2354	1.4468
C	-1.6052	-2.4757	-1.9882
C	-2.8014	-2.3443	-2.9521
C	-1.1273	-3.9337	-1.8258
H	-0.7725	-1.9566	-2.4819
H	-3.0380	-1.2835	-3.1047
H	-2.5415	-2.7841	-3.9246
H	-3.7019	-2.8456	-2.5820
H	-0.8517	-4.3368	-2.8097
H	-0.2401	-3.9671	-1.1819
H	-1.8837	-4.5928	-1.3888
H	-0.6742	6.8796	0.1938
H	-5.2400	-4.2731	0.9873
O	1.4397	-0.8152	0.6949
H	0.9542	1.8124	-0.6757
C	1.6890	-1.8137	1.7355
C	0.5724	-2.8558	1.7467
C	1.7787	-1.0560	3.0578
H	2.6394	-2.3125	1.5346
H	0.5078	-3.3831	0.7901
H	0.7654	-3.5923	2.5376
H	-0.3984	-2.3841	1.9419
H	2.5900	-0.3185	3.0317
H	0.8356	-0.5313	3.2543
H	1.9677	-1.7585	3.8795
C	2.0794	-0.7421	-0.6743
C	2.4912	-2.1385	-1.2023
C	4.2939	-0.3907	0.2300
H	1.7151	-2.8599	-0.9335
H	3.9804	-0.3030	1.2842
O	3.2377	0.0876	-0.6110
O	1.1605	-0.1479	-1.4232
C	2.7215	-2.1308	-2.7072
H	3.4755	-1.3796	-2.9695
H	3.0656	-3.1209	-3.0273
H	1.7858	-1.8861	-3.2183
C	4.6265	-1.8785	0.0377
O	3.7134	-2.6740	-0.5663
O	5.6402	-2.3749	0.4833
C	5.5134	0.4950	-0.0092
H	5.2494	1.5344	0.2097
H	6.3326	0.1792	0.6434
H	5.8363	0.4186	-1.0540

Free Energy -2639.053252 hartrees

Transition State $TS_{7\text{-}8L}$

Imaginary frequency at -41.3 cm^{-1}

Cartesian Coordinates

Zn	-0.7972	-0.9206	0.2206
S	-1.6312	0.5579	2.0432

N	-2.6224	-0.9775	-0.4487
C	-3.3329	0.1464	1.6003
C	-3.6262	-0.5512	0.3690
C	-4.3349	0.5700	2.4798
H	-4.0457	1.0768	3.3993
C	-5.0235	-0.7278	0.1147
H	-5.3362	-1.2324	-0.7888
C	-1.5462	2.3273	1.4423
H	-2.1758	2.8962	2.1370
H	-0.5040	2.6406	1.5715
C	-5.6843	0.3637	2.1920
H	-6.4563	0.6955	2.8815
C	-6.0072	-0.2881	0.9933
S	-0.8387	2.0587	-1.3071
N	1.9051	1.9410	0.1201
C	0.3870	3.3755	-1.1342
C	1.6085	3.1670	-0.4201
C	0.1100	4.6137	-1.7276
H	-0.8217	4.7297	-2.2786
C	2.4912	4.2730	-0.3239
H	3.4279	4.1587	0.2069
C	-2.0341	2.5532	0.0165
H	-2.2416	3.6201	-0.1251
H	-2.9541	1.9910	-0.1774
C	0.9937	5.6889	-1.6147
H	0.7609	6.6449	-2.0772
C	2.1860	5.5009	-0.9042
C	2.9264	1.6078	1.1259
C	2.8265	2.4594	2.4028
C	4.3510	1.5667	0.5471
H	2.6794	0.5799	1.3995
H	1.8134	2.4005	2.8207
H	3.5310	2.0745	3.1511
H	3.0648	3.5133	2.2219
H	4.3776	0.9255	-0.3420
H	4.7269	2.5584	0.2719
H	5.0330	1.1449	1.2972
C	-2.8540	-1.6183	-1.7588
C	-3.5434	-0.7017	-2.7887
C	-3.4853	-3.0228	-1.6716
H	-1.8460	-1.7826	-2.1612
H	-4.5877	-0.4872	-2.5411
H	-3.0039	0.2517	-2.8526
H	-3.5206	-1.1822	-3.7762
H	-3.4466	-3.5032	-2.6589
H	-2.9174	-3.6388	-0.9631
H	-4.5296	-3.0051	-1.3445
H	2.8968	6.3197	-0.8047
H	-7.0510	-0.4645	0.7356
O	2.5820	-1.7083	0.9845
H	1.1846	1.2427	0.0063
C	3.2219	-2.4365	2.0782
C	3.4254	-1.4593	3.2329
C	4.5345	-3.0289	1.5703
H	2.5455	-3.2374	2.4068
H	2.4638	-1.0467	3.5589
H	3.8991	-1.9732	4.0796
H	4.0786	-0.6356	2.9179
H	4.3590	-3.7089	0.7291
H	5.2003	-2.2243	1.2321
H	5.0343	-3.5819	2.3767
C	1.3663	-2.2701	0.5198
C	1.6057	-3.5065	-0.3534
C	1.6703	-0.9669	-1.7759
H	2.0836	-4.2452	0.2985
H	2.2234	-0.0275	-1.6610
O	0.8918	-1.1694	-0.5679
O	0.3659	-2.3221	1.3454
C	0.3470	-4.0917	-0.9746
H	-0.1578	-3.3520	-1.6044
H	0.6009	-4.9673	-1.5823
H	-0.3383	-4.3913	-0.1756
C	2.7207	-2.0648	-2.0101
O	2.6240	-3.2589	-1.3878
O	3.6150	-1.8746	-2.8072
C	0.7379	-0.8940	-2.9875
H	-0.0144	-0.1172	-2.8247
H	1.3242	-0.6438	-3.8779
H	0.2350	-1.8539	-3.1510

Free Energy: -2639.044907 hartrees

Compound 8_L

Cartesian Coordinates

Zn	1.1371	-0.7053	-0.3308
S	2.6385	0.7451	-1.6634
N	2.7164	-1.5058	0.4596
C	4.0900	-0.0807	-0.9738
C	3.9486	-1.1149	0.0261
C	5.3379	0.3293	-1.4564
H	5.3752	1.1022	-2.2227
C	5.1901	-1.6525	0.4931

H	5.1801	-2.4273	1.2469
C	2.5548	2.2759	-0.5977
H	3.4474	2.8656	-0.8371
H	1.6620	2.8159	-0.9315
C	6.5209	-0.2277	-0.9700
H	7.4856	0.0987	-1.3500
C	6.4219	-1.2231	0.0123
S	0.9180	1.2522	1.4871
N	-1.0541	1.9691	-0.9012
C	-0.1819	2.6760	1.2702
C	-0.9718	2.8997	0.0964
C	-0.1717	3.6058	2.3198
H	0.4190	3.3843	3.2070
C	-1.6470	4.1496	0.0296
H	-2.2418	4.3777	-0.8446
C	2.5130	1.9945	0.8989
H	2.6523	2.9295	1.4529
H	3.2971	1.2857	1.1883
C	-0.8727	4.8089	2.2363
H	-0.8475	5.5206	3.0577
C	-1.5974	5.0735	1.0671
C	-1.6816	2.1095	-2.2268
C	-1.0288	3.2145	-3.0724
C	-3.2192	2.2061	-2.1989
H	-1.4513	1.1517	-2.7037
H	0.0561	3.0588	-3.1269
H	-1.4361	3.1772	-4.0907
H	-1.2129	4.2179	-2.6721
H	-3.6363	1.3681	-1.6312
H	-3.5835	3.1422	-1.7610
H	-3.5983	2.1409	-3.2269
C	2.5013	-2.5160	1.5181
C	3.0271	-2.0878	2.9030
C	2.9252	-3.9448	1.1207
H	1.4085	-2.5629	1.6319
H	4.1193	-2.0326	2.9479
H	2.6234	-1.1004	3.1606
H	2.6916	-2.8091	3.6603
H	2.5612	-4.6560	1.8746
H	2.4806	-4.2069	0.1521
H	4.0098	-4.0649	1.0383
H	-2.1469	6.0079	0.9645
H	7.3251	-1.6817	0.4134
O	-4.0057	-1.7898	-0.1672
H	-0.8899	0.9885	-0.6683
C	-4.0385	-1.2250	1.2073
C	-4.5626	0.1938	1.0629
C	-2.6253	-1.2843	1.7768
H	-4.7244	-1.8442	1.7959
H	-5.5473	0.2016	0.5809
H	-4.6538	0.6415	2.0602
H	-3.8680	0.8015	0.4747
H	-2.3098	-2.3196	1.9426
H	-1.9338	-0.8054	1.0747
H	-2.5902	-0.7452	2.7307
C	-3.8915	-3.1185	-0.2903
C	-3.6212	-3.5761	-1.7360
C	-1.1262	-2.0459	-1.6101
H	-2.7440	-4.2323	-1.6804
H	-1.5575	-2.5451	-0.7290
O	-0.4958	-0.8594	-1.2353
O	-3.9737	-3.9307	0.6124
C	-4.8153	-4.3611	-2.2804
H	-5.6920	-3.7077	-2.3589
H	-4.5720	-4.7561	-3.2724
H	-5.0417	-5.1921	-1.6043
C	-2.2521	-1.7085	-2.6207
O	-3.3724	-2.5022	-2.6734
O	-2.1745	-0.8081	-3.4236
C	-0.1753	-3.0485	-2.3002
H	0.6115	-3.3412	-1.5930
H	-0.7019	-3.9571	-2.6237
H	0.2842	-2.5729	-3.1749

Free Energy: -2639.071907 hartre
